# RNA binding protein TIAR modulates HBV replication by tipping the balance of pgRNA translation

**DOI:** 10.1038/s41392-023-01573-7

**Published:** 2023-09-13

**Authors:** Ting Zhang, Huiling Zheng, Danjuan Lu, Guiwen Guan, Deyao Li, Jing Zhang, Shuhong Liu, Jingmin Zhao, Ju-Tao Guo, Fengmin Lu, Xiangmei Chen

**Affiliations:** 1 Department of Microbiology and Infectious Disease Center, School of Basic Medical Sciences, Peking University Health Science Center, Beijing, 100191 China; 2 Department of Pathology and Hepatology, The Fifth Medical Center of Chinese PLA General Hospital, Beijing, 100039 China; 3 Department of Experimental Therapeutics, Baruch S. Blumberg Institute, Doylestown, PA 18902 USA; 4 Beijing Key Laboratory of Hepatitis C and Immunotherapy for Liver Diseases, Peking University Hepatology Institute, Peking University People’s Hospital, Beijing, 100044 China

**Keywords:** Microbiology, Cell biology

## Abstract

The pregenomic RNA (pgRNA) of hepatitis B virus (HBV) serves not only as a bicistronic message RNA to translate core protein (Cp) and DNA polymerase (Pol), but also as the template for reverse transcriptional replication of viral DNA upon packaging into nucleocapsid. Although it is well known that pgRNA translates much more Cp than Pol, the molecular mechanism underlying the regulation of Cp and Pol translation efficiency from pgRNA remains elusive. In this study, we systematically profiled HBV nucleocapsid- and pgRNA-associated cellular proteins by proteomic analysis and identified TIA-1-related protein (TIAR) as a novel cellular protein that binds pgRNA and promotes HBV DNA replication. Interestingly, loss- and gain-of-function genetic analyses showed that manipulation of TIAR expression did not alter the levels of HBV transcripts nor the secretion of HBsAg and HBeAg in human hepatoma cells supporting HBV replication. However, Ribo-seq and PRM-based mass spectrometry analyses demonstrated that TIAR increased the translation of Pol but decreased the translation of Cp from pgRNA. RNA immunoprecipitation (RIP) and pulldown assays further revealed that TIAR directly binds pgRNA at the 5’ stem-loop (ε). Moreover, HBV replication or Cp expression induced the increased expression and redistribution of TIAR from the nucleus to the cytoplasm of hepatocytes. Our results thus imply that TIAR is a novel cellular factor that regulates HBV replication by binding to the 5’ ε structure of pgRNA to tip the balance of Cp and Pol translation. Through induction of TIAR translocation from the nucleus to the cytoplasm, Cp indirectly regulates the Pol translation and balances Cp and Pol expression levels in infected hepatocytes to ensure efficient viral replication.

## Introduction

Hepatitis B virus (HBV) infection is the leading cause of chronic hepatitis B (CHB), cirrhosis, and hepatocellular carcinoma (HCC) worldwide. Approximately 296 million people lived with chronic HBV infection worldwide in 2019, as estimated by World Health Organization.^1^ The current standard of care medication for CHB includes nucleos(t)ide analogs (NAs), which are reverse transcription inhibitors, and pegylated interferon alpha (Peg-IFN-α), which regulates antiviral immune responses. Though NAs and Peg-IFN-α can efficiently suppress HBV replication and decrease the incidence of end-stage liver diseases, including HCC, none of them can induce a functional cure of CHB in the vast majority of treated patients.^2,3^ Recent studies have shown that some new therapies can improve clinical outcomes of CHB patients and increase the functional cure rate, such as the combined therapy with NA and antisense oligonucleotides (ASO), sequential IL-2 treatment after IFN-α therapy.^4,5^ However, novel therapeutics that can functionally cure CHB with limited treatment duration are still unmet medical needs.

HBV is a member of the *Hepadnaviridae* family and contains a 3.2 kb, partially double-stranded, relaxed circular DNA (rcDNA) genome with hepatocyte tropism and a very narrow host range.^6^ The virus infects hepatocytes by binding to its cellular receptor, sodium taurocholate cotransporting polypeptide (NTCP), and delivering viral nucleocapsid into the cytoplasm *via* endocytosis.^7^ The viral rcDNA genome in nucleocapsid is then transported into the nucleus and converted into episomal covalently closed circular (ccc) DNA to serve as the template for the transcription of viral RNA.^8,9^ Among the viral transcripts, the 3.5 kb pre-genomic RNA (pgRNA) plays a critical role in HBV replication, which not only acts as the translation template of core protein (Cp) and DNA polymerase (Pol) but also serves as the template for reverse transcriptional viral DNA synthesis.^6^ The binding of newly translated Pol to the stem-loop structure (ɛ) at the 5’ terminal region of pgRNA, primarily *in cis*,^10,11^ initiates the encapsidation of Pol-pgRNA complex by 120 Cp dimers and subsequent reverse transcriptional HBV DNA synthesis with concomitant degradation of the pgRNA templet by RNase H activity of Pol in the nucleocapsid.^12,13^ Apparently, the fact that each nucleocapsid is made of 240 Cp and one Pol protein implies that at least 240-fold more Cp over Pol molecules are required to support efficient HBV replication. Indeed, Cp is more efficiently translated than Pol in HBV-infected hepatocytes.^14^ Moreover, while the upstream Cp ORF is most likely translated by a cap-dependent ribosome scanning mechanism, the downstream Pol ORF is possibly translated by mechanisms involving leaky scanning and/or ribosome re-initiation.^14–17^ Nevertheless, it is conceivable that the translation of Cp and Pol from the bicistronic pgRNA ought to be tightly regulated by viral and host cellular factors to ensure efficient HBV replication in hepatocytes.

In order to decipher the molecular mechanism underlying pgRNA translation regulation, we hypothesized that the host cellular proteins regulating pgRNA translation and/or encapsidation may interact with pgRNA and be selectively packaged into nucleocapsids. Accordingly, we first profiled the cellular proteins that associate with purified nucleocapsids and the proteins that bind to pgRNA in the lysates of human hepatoma cells by mass spectrometry analyses. The comparison of the nucleocapsid-associated and pgRNA binding proteomes revealed 15 overlapped proteins that most likely interact with pgRNA and are co-packaged with pgRNA into nucleocapsids. Further functional and biochemical analyses demonstrated that an RNA binding protein, TIA-1 related protein (TIAR), specifically binds to the 5’ stem-loop (ɛ) structure of pgRNA to promote Pol translation and subsequent pgRNA packaging and viral replication. As our hypothesis predicted, TIAR is indeed co-packaged with pgRNA into nucleocapsids. TIAR is thus a novel host factor exploited by HBV to balance the relative expression of Cp and Pol from the bicistronic pgRNA to ensure efficient replication. Apparently, our findings open new perspectives for developing novel antiviral agents for CHB treatment.

## Results

### RNA-binding proteins are major cellular proteins associated with HBV nucleocapsids

**Fig. 1:**
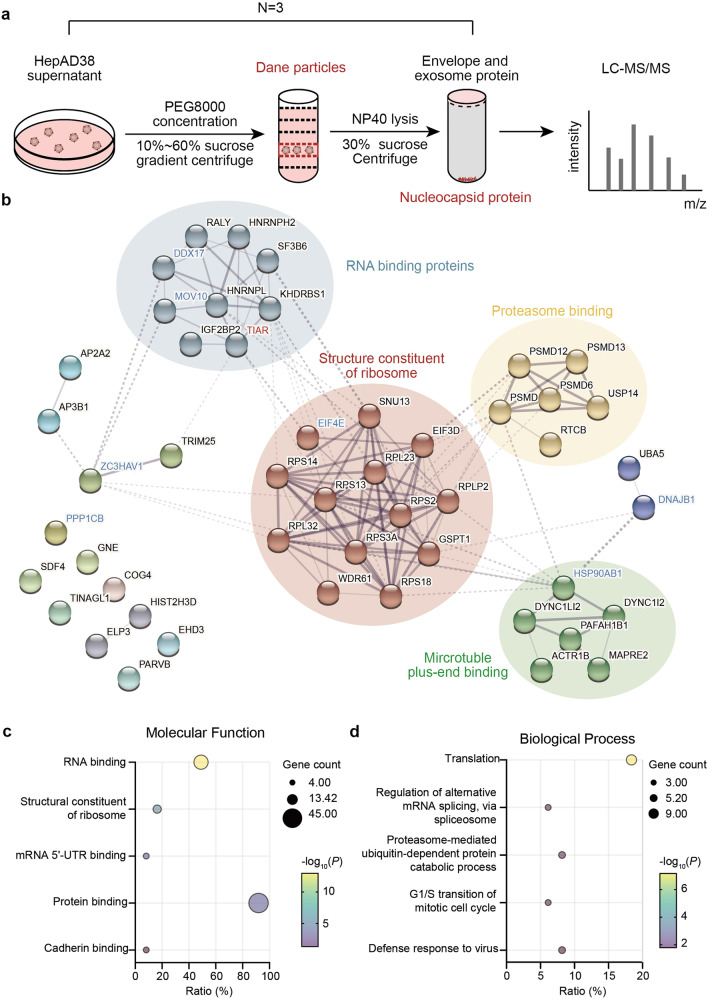
Identification of host factors associated with nucleocapsids. **a** Schematic presentation of experimental procedures for the identification of proteins associated with nucleocapsids. **b** PPI network of the 49 proteins associated with nucleocapsids based on STRING database. Proteins were clustered by the MCL algorithm (inflation parameter = 2.0). Clusters of functionally related nodes were manually encircled and annotated. Functional annotation determined by GO molecular function term in the STRING database. Proteins reported to be packaged in nucleocapsids were highlighted in blue. **c** Significantly enriched GO terms in molecular function (MF) identified by DAVID. **d** Significantly enriched GO terms in biological process (BP) identified by DAVID

To gain insight into the host factors that regulate HBV nucleocapsid assembly and reverse transcriptional DNA replication, we intended to identify cellular proteins associated with nucleocapsids. To achieve this goal, we separated HBV virion particles from subviral particles in the media of HepAD38 cells cultured in the absence of doxycycline (Dox) by 10%-60% sucrose gradient centrifugation. HBV DNA was detected in fractions 22 to 28 and peaked at fractions 25 and 26 overlapped with a minor peak of HBsAg, indicating that HBV Dane particles were enriched in those two fractions (supplementary Fig S1a).^18^ To obtain nucleocapsids and eliminate the exosomes co-sediment with virion particles, we treated the samples in fractions 25 and 26 with NP-40 and further pelleted by ultracentrifugation through a 30% sucrose cushion (Fig. 1a). Western blot and quantitative PCR (qPCR) assays demonstrated that HBV DNA and Cp were only detected in the pellet. In contrast, large envelope protein was predominantly detected in the supernatant (supplementary Fig S1b), indicating that nucleocapsids had been successfully obtained. The proteins in the supernatant and pellet were analyzed by liquid chromatography tandem mass spectrometry (LC-MS/MS). From three replicate experiments, 120 cellular proteins were identified in the pellets (supplementary Table S1), and 71 of those proteins were also present in the supernatants and thus not considered as nucleocapsid-associated cellular proteins. The remaining 49 proteins only detected in the pellets were considered as cellular proteins possibly associated with HBV nucleocapsids. Notably, several cellular proteins reported to be associated with HBV nucleocapsids in prior studies, such as HSP90,^19^ DDX17 and MOV10,^20,21^ were among these 49 proteins, which validates our experimental approach.

To investigate the functional associations of these proteins and their relationship to HBV replication, we constructed a protein-protein interaction (PPI) network with 49 genes (node genes) and 146 edges using the STRING v11 database (Fig. 1b).^22^ The results showed that those proteins had meaningfully more interactions than expected (146 vs. 48 expected edges, PPI enrichment *P* < 10^–16^). Markov Cluster Algorithm (MCL) analysis further revealed that ribosomal proteins and mRNA processing-associated RNA binders were the main clusters of the most interconnected nodes (PPI enrichment *P* < 10^–16^, respectively).^23^ In addition, proteasome complex (PPI enrichment *P* < 4.62$\times$10^–12^) and cytoplasmic dynein complex (PPI enrichment *P* < 2.82$\times$10^–10^) are also enriched in this network. Moreover, biological process Gene ontology (GO) term analysis revealed a strong enrichment linked to translation, mRNA splicing, and protein catabolic process (Fig. 1c, d). Notably, most of these enriched annotations are closely associated with RNA post-transcriptional processes.

### TIAR binds pgRNA and is packaged into nucleocapsids, but not empty capsids

**Fig. 2:**
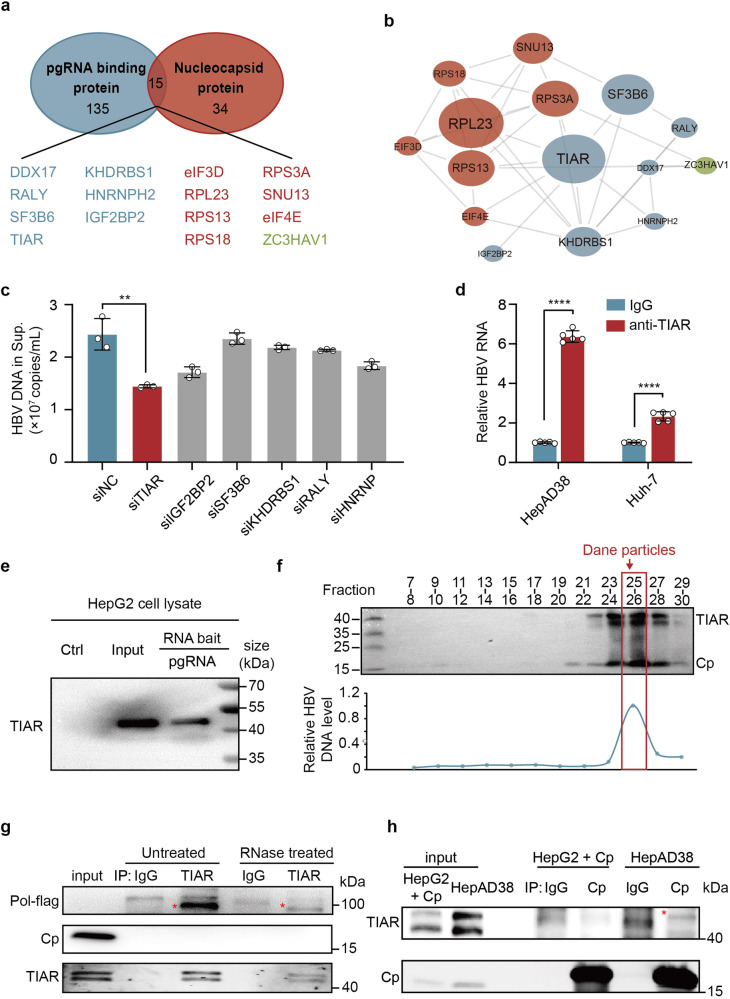
Validation of TIAR packaging into nucleocapsids and binding to pgRNA. **a** Comparison of pgRNA-interacting and nucleocapsid proteomes. **b** PPI network of the 15 common proteins between the two proteomes. Node size: the pulldown efficiency calculated by LC-MS/MS. **c** HepAD38 cells were transfected with indicated siRNA. HBV DNA levels in the culture supernatant were determined by qPCR (n = 3 per group). **d** Validation of TIAR-pgRNA interaction by RIP assay. pgRNA immunoprecipitated with anti-TIAR or mouse IgG from the cytoplasmic lysates of HepAD38 cells or Huh-7 cells transfected with prcccDNA/pCMV-Cre plasmids was quantified by RT-qPCR (n = 5 per group). Data are represented as mean ± SD, **P < 0.01, ****P < 0.0001. **e** Validation of TIAR-pgRNA interaction by RNA pulldown assay. HepG2 cell lysates were co-incubated with biotin-labeled full-length pgRNA transcribed in vitro. TIAR in the RNA-protein complex was pulled down by streptavidin beads and was detected by Western blot. Biotin-labeled Saccharomyces cerevisiae tRNA served as negative controls. **f** The culture supernatant of HepAD38 cells was separated by 10–60% linear sucrose density gradient centrifugation. The TIAR, Cp (top), and HBV DNA (bottom) levels in each fraction were detected by Western blotting and qPCR, respectively. **g** HepAD38 cells were transfected with Pol-flag plasmid. The cells were harvested 48 h post-transfection. IP followed by Western blot was performed with anti-TIAR antibodies and the immunoprecipitate was treated with or without RNase. Pol-flag was designated by asterisk. **h** HepG2 cells were transfected with pCMV-core plasmid. The cells were harvested 48 h post-transfection and lysed with NP40 lysis. Immunoprecipitation (IP) was performed with anti-core antibodies followed by Western blot analysis. HepAD38 cells served as positive control and TIAR packaged in nucleocapsids was designated by asterisks

Considering that Pol primarily binds to the pgRNA that translates itself to initiate the encapsidation of the Pol-pgRNA complex,^11^ we speculated that the host factors that bind to pgRNA and regulate Pol translation are most likely co-packaged with Pol-pgRNA complex into nucleocapsid. To cross-examine whether the identified nucleocapsid-associated cellular proteins bind pgRNA, biotin-labeled full-length pgRNA was used to pulldown pgRNA binding proteins from HepG2 cell lysates by using streptavidin magnetic beads and analyzed by mass spectrometry-based label-free quantitative proteomics.^24^ 150 cellular proteins were significantly associated with pgRNA (supplementary Table S2), and 15 of them overlapped with the nucleocapsid proteomes (Fig. 2a). Interestingly, the vast majority of these overlapping proteins (14, 93%) are ribosomal proteins (Fig. 2a, proteins in red) and RNA binders (Fig. 2a, proteins in blue) that are highly enriched in the cluster by MCL clustering. The relative abundance analysis of the proteins co-purified in pgRNA-protein complexes and PPI network analysis revealed that TIAR connected directly or indirectly with most of the others and efficiently interacted with pgRNA except for ribosomal proteins. Those results suggest that TIAR may play a critical role in the regulation of pgRNA metabolism and function and consequentially alter HBV replication (Fig. 2b). In the investigation of this hypothesis, we found that knockdown of the endogenous expression of TIAR, but not several other RNA binders, in HepAD38 cells significantly decreased the levels of HBV DNA in culture supernatants (Fig. 2c).

To further confirm the direct interaction between TIAR and pgRNA, we performed RNA immunoprecipitation (RIP), RNA pulldown and RNA Fluorescence in situ Hybridization (FISH) assays. The RIP assay showed that pgRNA could be immunoprecipitated with an antibody against TIAR from the lysates of HepAD38 cells or Huh-7 cells transfected with prcccDNA/pCMV-Cre plasmids (Fig. 2d). Consistently, pulldown of biotin-labeled pgRNA from HepG2 cell lysates also pulled down TIAR (Fig. 2e). The RNA-FISH combined with immunofluorescence (IF) assays also showed the colocalization of TIAR and HBV RNA in HepAD38 cells (supplementary Fig S2).

Next, we investigated whether TIAR was encapsidated in the nucleocapsids. First of all, sucrose gradient centrifugation analysis of HBV particles in the culture supernatant of HepAD38 cells demonstrated that TIAR co-sedimented with Cp and HBV DNA and thus most likely encapsidated in the nucleocapsids (Fig. 2f). In addition, a Co-immunoprecipitation (Co-IP) assay was conducted to assess the interaction between TIAR and Cp or Pol proteins in the HepAD38 cells transfected with pcDNA3.1-Pol-flag. The results showed that TIAR could interact with Pol but not Cp (Fig. 2g). Since both Pol and TIAR can bind to pgRNA, we treated immunoprecipitate with RNase. We found that TIAR interacted with Pol partly in an RNA-dependent manner. Finally, we conducted an IP assay with an antibody against HBV capsids in HepAD38 cells and HepG2 cells ectopically expressing HBV Cp. Western blot analysis of the capsid proteins showed that TIAR was detected in the capsids from HepAD38 cells, but not the empty capsids from HepG2 cells ectopically expressing HBV Cp (Fig. 2h). These results thus favor the hypothesis that TIAR is selectively packaged into nucleocapsids by binding to pgRNA, but not empty capsids.

### TIAR facilitates HBV replication *via* a post-transcriptional mechanism

**Fig. 3:**
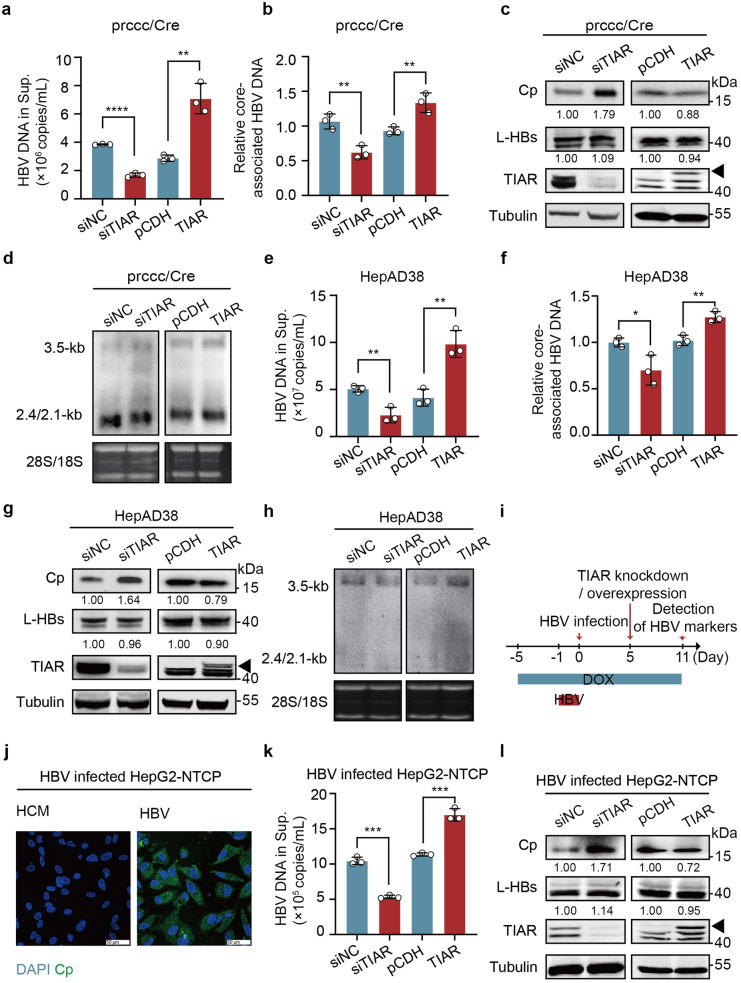
TIAR facilitates HBV replication *via* a post-transcriptional mechanism. **a**–**d** Huh-7 cells were co-transfected with prcccDNA and pCMV-Cre plasmids. After 6 h, cells were transfected with siTIAR or pCDH-TIAR-flag, respectively, siNC or pCDH as a negative control. The cells and culture supernatants were harvested 48 h post-transfection. (a) HBV DNA levels in the culture supernatants were determined by qPCR (n = 3 per group). **b** Core-associated HBV DNA levels were determined by qPCR (n = 3 per group). **c** Intracellular HBV proteins were analyzed by Western blot. α-Tubulin served as a loading control. The arrows represent TIAR-flag protein. **d** Intracellular HBV RNA levels were determined by Northern blot. Ribosomal RNA (28 S and 18 S) served as loading controls. **e**–**h** HepAD38 cells were transfected with siTIAR or pCDH-TIAR-flag with siNC or pCDH as a negative control, respectively. The levels of HBV DNA in the culture supernatants (n = 3 per group) (**e**), core-associated HBV DNA (**f**), intracellular proteins (n = 3 per group) (**g**), and intracellular HBV RNA (**h**) were analyzed 48 h post-transfection. **i** Schematic workflow of HBV infection experiments in HepG2-NTCP cells. **j** HBV Cp was detected in HBV-infected HepG2-NTCP cells by IF staining at 5 dpi. One representative experiment is shown. Scale bars: 50 µm. **k** In the HBV-infected HepG2-NTCP cells, HBV DNA levels in the culture supernatant were determined by qPCR (n = 3 per group). **l** Intracellular HBV proteins were analyzed by Western blot. Data are represented as mean ± SD, *P < 0.05, **P < 0.01, ***P < 0.001, ****P < 0.00001, ns, no significance

To investigate the role of TIAR in HBV replication, we determined HBV proteins and replication intermediates in Huh-7 cells transiently transfected with prcccDNA/pCMV-Cre plasmids that produce recombination cccDNA to launch HBV replication under the condition of down- or up-regulated TIAR expression.^25^ The results showed that small interfering RNA (siRNA) knockdown of TIAR expression significantly decreased the level of HBV DNA in the culture supernatant (Fig. 3a) and core-associated HBV DNA (Fig. 3b) but increased the level of intracellular Cp (Fig. 3c). On the contrary, over-expression of TIAR significantly increased the level of HBV DNA in culture supernatants and core-associated HBV DNA but repeatably decreased the level of intracellular Cp (Fig. 3a–c). To our surprise, neither knockdown nor overexpression of TIAR significantly altered the levels of secreted HBsAg and HBeAg (supplementary Fig S3a) and intracellular L-HBs (Fig. 3c). Moreover, alteration of TIAR expression also did not change the levels of HBV RNA transcripts (Fig. 3d, supplementary Fig S4a). Similar observation was also made in HepAD38 cells (Fig. 3e–h, supplementary Fig S3b&S4b) as well as HBV infected HepG2-NTCP cells (Fig. 3i-l, supplementary Fig S3c & S4c).

Taken together, these results suggest that TIAR facilitates HBV replication *via* a post-transcriptional mechanism.

### TIAR specifically regulates pgRNA translation

While no impact of TIAR on the levels of all the HBV transcripts and secreted HBsAg and HBeAg indicates that TIAR does not affect the transcription and stability of HBV RNA as well as the translation of pre-C RNA and 2.1/2.4 kb subgenomic RNA, TIAR altered the levels of HBV DNA and Cp in opposite directions (Fig. 3). Since both Cp and Pol are translated from pgRNA, and it was reported that the increased translation of Pol promoted HBV replication,^26^ we therefore speculated that TIAR might regulate pgRNA translation by tipping the balance of Cp and Pol expression.

**Fig. 4:**
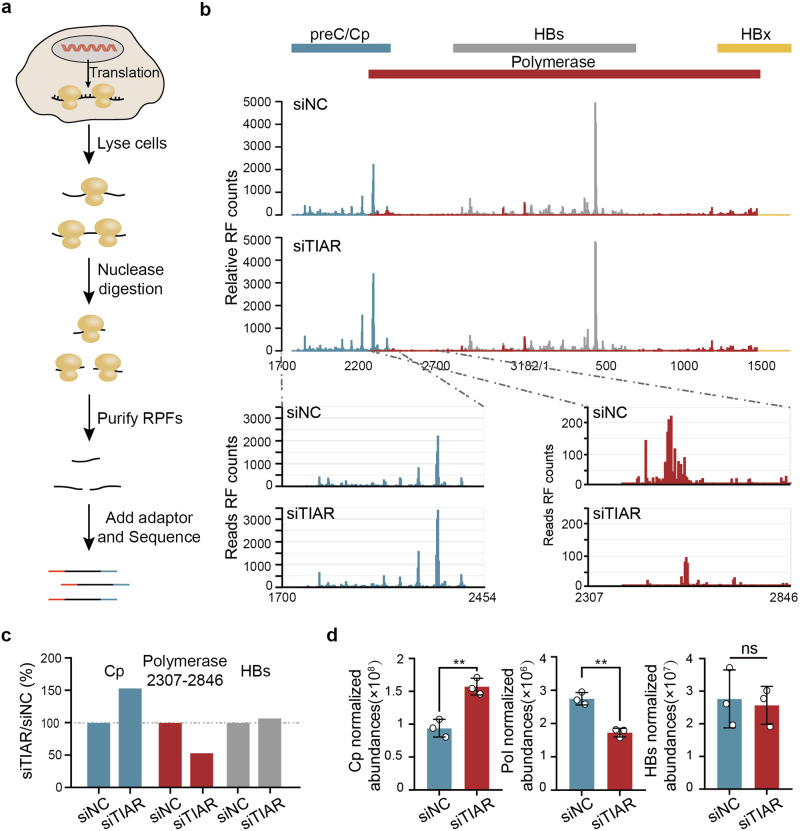
TIAR regulates the translation of bicistronic pgRNA. **a** The schematic workflow of Ribo-seq. **b** The relative RF counts of HBV ORFs in HepAD38 cells transfected with siTIAR or siNC were analyzed by RiboWave. **c** Relative RF abundance of Cp-ORF, Pol initiation region, and HBs-ORF. **d** The expression of Cp, Pol, and HBs in HepAD38 cells transfected with siTIAR or siNC was detected by PRM-based MS. Data are represented as mean ± SD (n = 3 per group), **P < 0.01, ns no significance

To test this hypothesis, we performed a ribosome profiling sequencing (Ribo-seq) in HepAD38 cells transfected with siTIAR or control siNC. The isolated polysomes were treated with ribonuclease to digest unprotected regions of RNA. The resulting ribosome-protected RNA fragments (or ribosome footprints, ~30 nucleotides) were used to generate a sequencing library (Fig. 4a).^27,28^ The quality of the library was verified by the results that the majority of ribosome footprints (RFs, Ribo-seq reads) were 27 to 31 nucleotides (nt) in length (supplementary Fig S5a), which is consistent with previous reports.^27,29^ Additionally, the RFs exhibited a three‐nucleotide periodicity,^29,30^ indicating the exact translating reading frame on mRNA (supplementary Fig S5b). Furthermore, the HBV transcripts had no distinguishable difference between HepAD38 cells transfected with siTIAR and siNC (supplementary Fig S5c), the RF count difference of HBV ORFs between the two treatment conditions should only reflect their difference in translation.

Because most ORFs of HBV are overlapped, RiboWave, a Ribo-seq data analysis tool, was used to precisely locate the RFs to the HBV genome by inferring the P-site of each RF.^31^ Compared with that in HepAD38 cells transfected with siNC, the RF counts of Cp ORF were higher in HepAD38 cells transfected with siTIAR. On the contrary, the RF counts of Pol ORF, particularly in the initial region of Pol ORF, were lower in HepAD38 cells transfected with siTIAR. The translation of HBs had no significant differences in HepAD38 cells transfected with siTIAR or control siNC, which is consistent with the results that neither knockdown nor overexpression of TIAR altered the level of secreted HBsAg (Fig. 3). Since the counts of HBx ORF were very low, the effect of siRNA knockdown of TIAR expression on the translation of HBx could not be confidently determined (Fig. 4b, c). To further evaluate the role of TIAR in pgRNA translation, a parallel reaction monitoring (PRM)-based MS was performed in HepAD38 cells transfected with siTIAR or control siNC. Similar to the results obtained from Ribo-seq analysis, TIAR knockdown significantly increased the abundance of Cp but decreased the abundance of Pol. In contrast, the abundance of HBs was not affected (Fig. 4d). In summary, the results presented herein strongly suggest that TIAR facilitates HBV replication by tipping the balance of Cp and Pol translation from pgRNA in favor of Pol expression, which promotes pgRNA encapsidation and consequentially enhances viral DNA replication and progeny virion production.

### TIAR regulates pgRNA translation *via* binding to its 5’ ε element

**Fig. 5:**
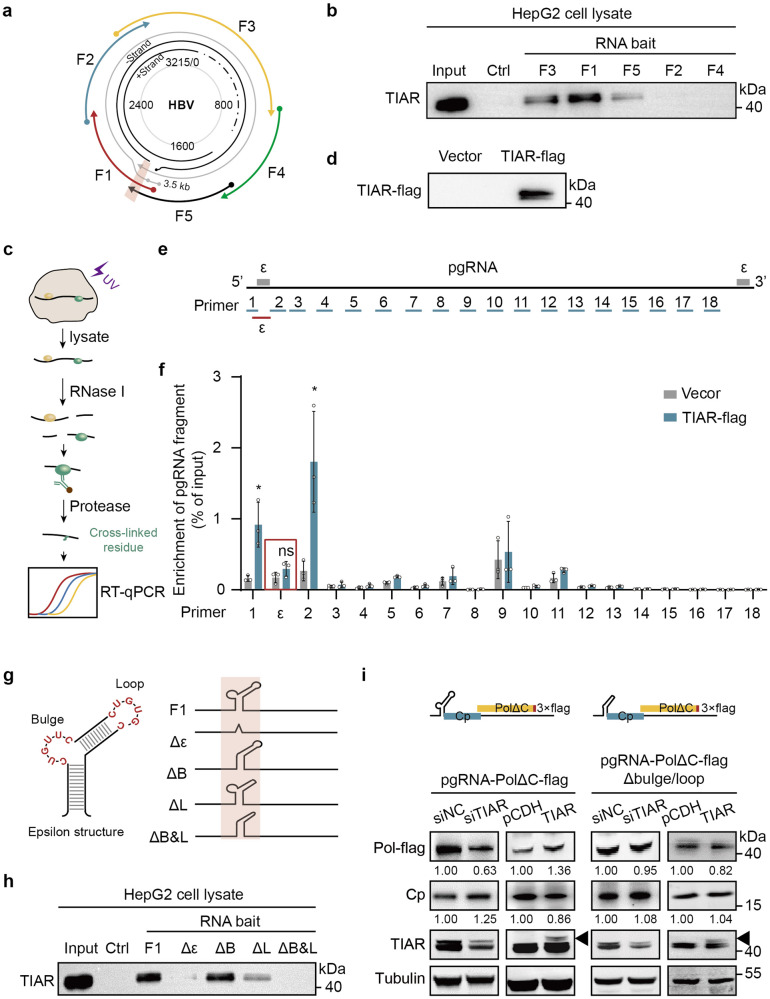
TIAR specifically binds to the bulge and loop structures of 5’ epsilon element in pgRNA. **a** The schematic diagram of 5 RNA fragments of the HBV genome used in the pulldown assay. **b** HepG2 cell lysates were co-incubated with the indicated biotin-labeled RNA fragment (F1, F2, F3, F4 and F5) transcribed in vitro. TIAR in the RNA-protein complex was pulled down by streptavidin beads and detected by Western blot. Biotin-labeled Saccharomyces cerevisiae tRNA served as negative controls. **c** The schematic workflow of CLIP assay. **d** Western blot analysis was performed to verify the accuracy of the CLIP results by anti-flag. **e** pgRNA sequence was covered by primers 1 to 18. **f** HepAD38 cells were transfected with pCDH-TIAR-flag or pCDH-flag plasmid. CLIP followed by RT-qPCR was performed to clarify the sequence of pgRNA that bound to TIAR. Data are represented as mean ± SD (n = 3 per group), *P < 0.05, ns, no significance. **g** The schematic diagram of 5’ ɛ region of pgRNA and derived F1 RNA fragments with the indicated deletion of RNA structure element in the 5’ ɛ region. **h** HepG2 cell lysates were co-incubated with biotin-labeled F1 or the indicated F1- derived fragments (F1, ∆ε, ∆B, ∆L, ∆B&L) transcribed in vitro. TIAR in the RNA-protein complex pulled down by streptavidin beads was detected by Western blot. **i** Huh-7 cells were co-transfected with pcDNA3.1-pgRNA-PolΔC-flag or the mutant plasmid, and siTIAR or pCDH-TIAR-flag. Intracellular PolΔC-flag and Cp were detected by Western blot

To understand the mechanism underlying TIAR regulation of pgRNA translation, we mapped the binding site of TIAR in pgRNA. Specifically, five biotin-labeled overlapping HBV RNA fragments (F1 to F5) covering the entire pgRNA were synthesized by in vitro transcription (Fig. 5a) and incubated with HepG2 cell lysates. Western blot analysis of the RNA-bound proteins showed that TIAR more efficiently bound to F1 and less efficiently bound to F3 and F5 (Fig. 5b). To further explore the exact binding site of TIAR in pgRNA, we conducted the cross-linking and immunoprecipitation, and qPCR (CLIP-qPCR) assays. The quality control of the experiment showed that the specific antibody captured the TIAR-flag protein (Fig. 5c-d). Then, a series of primers (1–18) was designed to cover the pgRNA sequence (Fig. 5e). CLIP-qPCR showed that TIAR potentially bound to the target regions of primer 1 and 2 (Fig. 5f), both contained in F1 fragment. We additionally designed another primer (primer ε) between target regions of primer 1 and 2 to further specify the binding sequence. Interestingly, the target sequence of primer ε pulled down by anti-TIAR could not be obviously detected (Fig. 5f, red) due to the residual amino acids would halt reverse transcriptase,^32^ suggesting that TIAR might bind to the ε element of pgRNA.

By analyzing the sequences of the ε structure of pgRNA, we found that there are two putative TIAR recognition and binding sequence motifs, a CUGUUC (nt1862–1867) sequence in the lower bulge of ε element and a CUGUGC sequence in the upper loop of ε element (Fig. 5g, left).^33,34^ Consequently, we speculated that TIAR might bind to the 5’ ε element of pgRNA to regulate its translation. To test this hypothesis, we synthesized biotin-labeled F1-derived RNA fragments with the deletion of the entire ε element, lower bulge and/or high loop and incubated them with HepG2 cell lysate (Fig. 5g, right). In support of our hypothesis, the deletion of ε element or bulge and loop structures abolished TIAR binding, and deletion of bulge or loop reduced TIAR binding (Fig. 5h). These results thus indicate that TIAR binds to pgRNA mainly *via* interaction with the bulge and loop structures of 5’ ε element.

To directly determine the effect of TIAR on Cp and Pol translation from pgRNA, we constructed a plasmid expressing truncated pgRNA with wild-type or bulge and loop deleted 5’ ɛ element and encoding a full-length Cp, but a C-terminally truncated Pol with 3$\times$ flag-tag (pcDNA3.1-pgRNA-PolΔC-flag). The results presented in Fig. 5i demonstrated that TIAR knockdown significantly reduced the expression of PolΔC-flag and increased the expression of Cp in Huh-7 cells transfected with the plasmid expressing truncated pgRNA with wild-type, but not the mutant 5’ ɛ element. Besides, TIAR overexpression also showed consistent results (Fig. 5i). These results strongly suggest that TIAR selectively regulates the translation of pgRNA by binding to the bulge and loop structures of pgRNA 5’ ε element.

### HBV replication induces the cytoplasmic translocation of TIAR

**Fig. 6:**
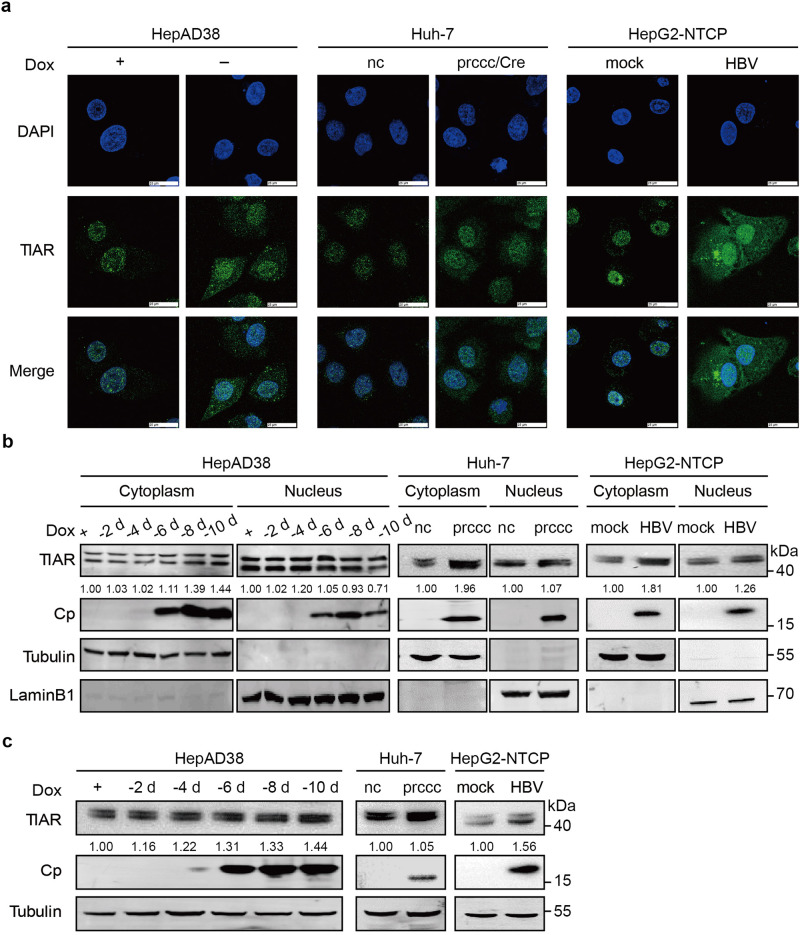
HBV modulates the subcellular localization of TIAR. **a** HepAD38 cells (left), Huh-7 cells transfected with prcccDNA/ pCMV-Cre plasmids (middle), or HBV-infected HepG2-NTCP cells (right) were fixed and subjected to IF staining. Nuclei were stained with DAPI. Scale bars: 25 µm. **b** TIAR protein levels in the cytoplasm and nucleus of HepAD38 cells (left), Huh-7 cells transfected with prcccDNA/pCMV-Cre plasmids (middle) or HBV infected HepG2-NTCP cells (right) were detected by Western blot. **c** Total TIAR levels in HepAD38 cells (left), Huh-7 cells transfected with prcccDNA/ pCMV-Cre plasmids (middle) or HBV infected HepG2-NTCP cells (right) were detected by Western blot

It has been reported that TIAR localizes predominantly in the nuclei of cells. However, under the selected stress conditions including virus infection, TIAR undergoes dramatic nuclear to cytoplasmic translocation.^35^ In order to investigate whether HBV infection affects the subcellular localization of TIAR, we performed an IF assay, which clearly showed the cytoplasmic accumulation of TIAR in HepAD38 cells upon the induction of HBV replication (Fig. 6a, left). A similar observation was also made in Huh-7 cells transfected with prcccDNA/pCMV-Cre plasmids (Fig. 6a, middle) and HBV-infected HepG2-NTCP cells (Fig. 6a, right). The accumulation of TIAR in the cytoplasm was also evaluated by cell fractionation, in HepAD38, Huh-7, and HepG2-NTCP cells (Fig. 6b). Interestingly, we also observed that the levels of TIAR in the nucleus seem no noticeable change and the total TIAR levels increase after HBV replication (Fig. 6c). However, HBV replication had no effect on TIAR RNA (supplementary Fig S6a-b). Since TIAR predominantly localizes in the nucleus in the resting state, the increase of TIAR in the cytoplasm indicates that HBV infection and replication induce the TIAR translocation from the nucleus into the cytoplasm of hepatocytes.

### Cp regulates the subcellular distribution of TIAR

**Fig. 7:**
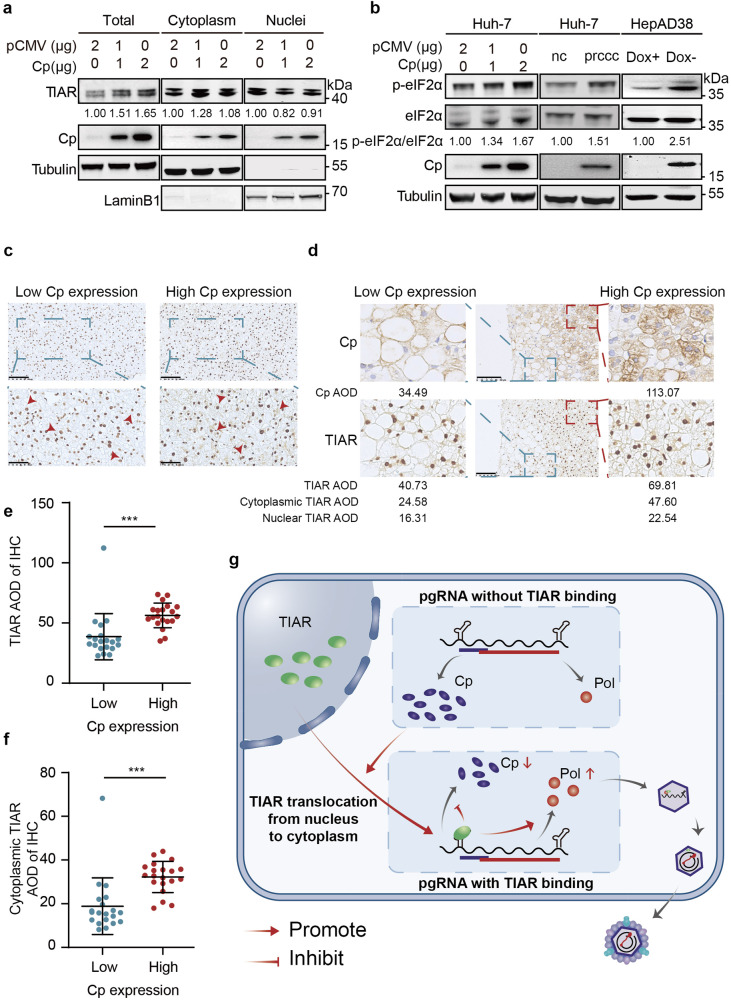
Cp modulates subcellular localization of TIAR. **a** TIAR protein levels in the cytoplasm and nucleus were detected by Western blot in Huh-7 cells transfected with 0, 1, 2 µg pCMV-Cp plasmids. **b** Huh-7 cells were co-transfected with 0, 1, 2 µg pCMV-Cp and 2, 1, 0 µg vector (left), HepAD38 cells were cultured with Doxycycline or not (middle), and Huh-7 were co-transfected with prcccDNA and pCMV-Cre plasmids (right), total and phosphorylation levels of eIF2α were detected by Western blot. **c** The representative IHC results of TIAR in liver tissues of CHB patients with different Cp expression. Arrows represent hepatocytes where TIAR is predominantly localized in the nucleus (left) or cytoplasm (right). Scale bar: 100 μm (top) and 50 μm (bottom). **d** TIAR subcellular localization in cells with different Cp expression in the same sample. Scale bar: 100 μm. The AOD of Cp and TIAR was analyzed by ImageJ. **e** TIAR AOD value of 40 liver biopsy samples analyzed by ImageJ (n = 20 per group). **f** Cytoplasmic TIAR AOD value of 40 liver biopsy samples analyzed by ImageJ (n = 20 per group). Data are represented as mean ± SD, ***P < 0.001. (**g**) Graphical Abstract

To investigate the role of individual viral proteins in the alteration of TIAR subcellular distribution in HBV replicating hepatocytes, we examined the subcellular localization of TIAR in Huh-7 cells ectopically expressing Cp, Pol, HBx, or HBs. Interestingly, while over-expression of all the other viral proteins did not affect TIAR subcellular distribution (supplementary Fig S7a-c), expression of Cp decreased TIAR in the nuclear fraction but increased the abundance of TIAR in the cytoplasmic fraction in a dose-dependent manner (Fig. 7a). On the contrary, prcccDNA with a Cp mutation could not increase the cytoplasmic distribution of TIAR (supplementary Fig S7d). However, the expression of Cp did not apparently alter the levels of TIAR RNA (supplementary Fig S6c). These results indicated that Cp expression up-regulates TIAR expression and induce TIAR to accumulate in the cytoplasm.

It has been reported that the phosphorylation of eIF2α could lead to the cytoplasmic accumulation of TIAR.^36^ To investigate whether eIF2α is involved in the nuclear/cytoplasmic redistribution of TIAR, we detected the phosphorylation level of eIF2α. We found that both Cp expression and HBV replication could increase the phosphorylation level of eIF2α (Fig. 7b), suggesting that HBV replication and Cp expression might modulate TIAR subcellular localization *via* eIF2α phosphorylation.

To further investigate the role of Cp in the subcellular localization of TIAR in CHB patients, we employed immunohistochemistry (IHC) to examine the relationship between Cp expression and TIAR localization in the liver biopsy from 40 patients with CHB. We found that TIAR mainly distributed in the cytoplasm in the liver tissues with high Cp expression, while it was presented primarily in the nuclei of hepatocytes in the liver tissues with low Cp expression (Fig. 7c). In addition, we compared TIAR subcellular localization in cells with different Cp expression in the same sample. As highlighted in Fig. 7d and supplementary Fig S7e, TIAR cytoplasmic distribution was higher in the cells with high Cp expression. Besides, we also quantitatively analyzed the results of IHC and found that the average optical density (AOD) of TIAR in liver biopsies with high Cp expression was significantly higher than that with low Cp expression (Fig. 7e), especially in the cytoplasm (Fig. 7f, supplementary Fig S7f). Moreover, both total and cytoplasmic TIAR levels were weakly positively correlated with HBV viral load (supplementary Fig S7g-h). These IHC results further support the notion that the HBV core protein induces the cytoplasmic accumulation of TIAR to favor the translation of Pol, which subsequently promotes pgRNA packaging and viral DNA replication.

## Discussion

HBV pgRNA is a bicistronic mRNA that translates Cp and Pol in the canonical cap-dependent manner.^37^ As two viral proteins essential for pgRNA encapsidation and viral DNA replication, the balanced translation of Cp and Pol ought to be critical for the efficient replication of HBV. It should thus be tightly regulated by viral and host factors. In this study, we obtained several lines of evidence supporting the notion that TIAR, an RNA binding protein, specifically binds to the 5’ ɛ structure of pgRNA to shift the balance of Cp and Pol translation in favor of Pol translation, which subsequently enhances the pgRNA encapsidation, DNA replication, and virion production in HBV-infected hepatocytes (Figs. 1–5). Similar to several other cellular proteins that regulate pgRNA encapsidation and viral DNA synthesis, such as HSP90,^19^ DDX17,^20^ APOBEC3G,^38^ and protein phosphatase 1 (PP1),^39^ TIAR is also co-packaged into the nucleocapsids (Fig. 1) but not empty capsids (Fig. 2h). In agreement with its specific regulation of pgRNA translation, experimental modulation of TIAR expression in hepatocytes does not alter the levels of viral RNA as well as secreted HBsAg and HBeAg (Fig. 3 and supplementary Figs 3–4.

TIAR is initially discovered as one of the main components of cytotoxic T lymphocyte granules.^40^ However, further studies revealed that TIAR is one of the ubiquitously expressed RNA-binding proteins involved in forming stress granules in the cytoplasm of cells in response to diverse stimuli, including viral infection.^36^ Two isoforms of TIAR are generated by the alternative splicing of the pre-mRNAs,^41^ *i.e*., 42-kDa TIARα and 40-kDa TIARβ. While the nuclear TIAR regulates transcription and pre-mRNA splicing,^33,42–44^ the cytoplasmic TIAR modulates the stability and translation of mRNAs.^45–48^ Recently, TIAR was reported to modulate the replication of several medically necessary viruses, such as human immunodeficiency virus 2 (HIV-2),^49^ West Nile Virus (WNV), and hepatitis C virus (HCV).^50–52^ In this study, we demonstrate that both TIAR isoforms can be packaged into HBV nucleocapsids (supplementary Fig S2), suggesting that both the isoforms can bind and regulate the translation of pgRNA and HBV replication.

Mechanistically, it has been reported that TIAR binds viral RNA to regulate the encapsidation of HIV genomic RNA and the release of HCV infectious virion particles.^49,52^ In this study, we demonstrated that TIAR directly binds to the 5’ ε of pgRNA (Fig. 5). Using Ribo-seq assay and PRM-based MS, we showed that knockdown of TIAR reduced the translation initiation of Pol while increasing the translation of Cp. Those results imply that binding TIAR to pgRNA at the 5’ ε increases Pol translation, but suppresses Cp translation. A possible interpretation of this phenomenon is that the binding of TIAR to pgRNA at the 5’ ε may either disturb the formation of the eIF4F complex on the 5’ cap of pgRNA or interfere with the assembly of 80 S complex assembly at the AUG codon of Cp ORF to reduce Cp translation but favor the translation of Pol. Currently, there are two hypothetical models on the mechanism of Pol translation, *i.e*., leaky scanning and ribosome re-initiation.^15^ The leaky scanning model predicts that the scanning complex bypasses AUG codons if the surrounding nucleotide context is suboptimal and continues scanning until encountering downstream AUG to initiate translation.^53^ However, there are five AUG codons upstream of the authentic Pol initiation codon.^14^ Thus, the scanning complex is unlikely to bypasse all the five AUG codons to translate Pol. On the contrary, the ribosome re-initiation model predicts that ribosomes terminate translation of the short upstream ORF (uORF) and then reinitiate translation of downstream ORF.^14–16,37,54^ In addition to Cp-ORF, there are two short ORFs, C0-ORF and J-ORF,^14–16^ preceding Pol-ORF. In our Ribo-seq results, there is no apprant difference in RF counts of C0-ORF. Interestingly, the RF counts of J-ORF decreased after TIAR knockdown (about 4 folds, data not shown). J-ORF is reported to be the uORF that may trigger ribosome re-initiation and favor the translation of Pol. Therefore, our data indicate that TIAR’s upregulation of Pol translation by TIAR may be related to the increased J-ORF translation. Further mechanistic studies are required to confirm this hypothesis.

Besides, cellular proteins that quantitatively regulate Cp and Pol translation, Pol protein itself has also been shown to inhibit pgRNA translation, primarily by promoting the encapsidation of pgRNA. Examining whether other viral proteins can also regulate the pgRNA translation will be interesting. The results presented in Figs. 6–7, and supplementary Fig S7 clearly demonstrated that expression of Cp, but not Pol, HBs, or HBx, increased the amount of TIAR in the cytoplasm. The similar redistribution of TIAR proteins was also observed in HBV replicating hepatocytes in vitro (Fig. 6) and in vivo in the livers of chronic HBV carriers (Fig. 7). Those results imply that Cp may indirectly regulate pgRNA translation *via* induction of TIAR cytoplasmic accumulation, which finetunes the quantitative balance of Cp and Pol translation as well as pgRNA packaging and translation to ensure the efficient HBV replication in hepatocytes (Fig. 7g). Apparently, therapeutic disruption of this feedback regulation loop will inhibit HBV replication and facilitate the cure of CHB.

It has been reported that persistent HBV infection and replication can accelerate the development of human liver fibrosis and HCC. In this study, we demonstrated that HBV replication and Cp expression could increase TIAR expression (Figs. 6 and 7), and TIAR levels were positively correlated with HBV viral load (supplementary Fig S7f-g). However, whether TIAR is involved in the progression of human liver fibrosis and HCC remains unclear. We discovered that TIAR expression is higher in tumor tissues of HCC, and its high expression is associated with poor prognosis of HCC (supplementary Fig S8a-d) but is not related to the staging of liver fibrosis (supplementary Fig S8e-f) by using public databases.^55–58^ Nevertheless, the occurrence of HCC and cirrhosis is a complex process, which is not only related to HBV replication but also to repeated inflammatory injury. Additionally, TIAR is first identified as a cytotoxicity T cell granule component and whether increased TIAR in HCC tissues affects CD8 + T cell function, and then affects the progression of HCC is unclear. Therefore, the potential mechanism for how TIAR promotes HCC progression is worthy to explore in the future.

In this study, we isolated proteins associated with nucleocapsids by sucrose density centrifugation. However, it is still possible that large protein complexes with the same sedimentation coefficient might cause false signals. Therefore, the other nucleocapsid-associated proteins identified in this study should be further experimentally validated in the future. Besides, we used RNA pulldown followed by LC-MS/MS assays to identify the cellular proteins binding to pgRNA. Several pgRNA interactive proteins previously reported were also identified, including DDX17,^20^ IGF2BP3,^59^ and ZC3HAV1.^60^ However, we failed to identify some reported pgRNA binding proteins, such as RBM24,^61^ RBM38,^62^ and CRM1,^63^ which might arise due to the relatively weak efficiency of the RNA pulldown assay.

In summary, this study identified TIAR as a novel cellular protein that binds pgRNA to facilitate DNA polymerase translation and consequentially promotes Pol-pgRNA complex packaging into nucleocapsids and HBV replication. Our findings shed new light on the molecular mechanism of HBV-host cell interaction and define a novel therapeutic target for chronic hepatitis B.

## Methods

More detailed procedures are provided in the Supplementary.

### liver biopsy samples

A total of 40 liver biopsy samples of patients with CHB were collected from The Fifth Medical Center of Chinese PLA General Hospital. The clinical background of the patients enrolled in this study is summarized in supplementary Table S3. Approval of the study protocol was obtained from the Ethics Committee of The Fifth Medical Center of Chinese PLA General Hospital (number: KY-2022–1–4–1). Informed consent was obtained from all subjects prior to participation.

### sucrose density gradient centrifugation

Sucrose density gradient centrifugation was performed as previously described.^18^ The solution with 20 mM Tris-HCl (pH 7.4) (Coolaber), 140 mM NaCl and 1 mM EDTA (Solarbio) was used to prepare discontinuous sucrose density gradients (10, 20, 30, 40, 50 and 60%). Culture supernatant was laid on the sucrose gradient and centrifuged at 25,000 rpm for 15 h at 10 °C in a Beckman SW50.1 rotor (Beckman Coulter).

### RNA pulldown assay

RNA pulldown assays based on Streptavidin were performed as previously described.^64^ PCR-amplified DNA fragments with T7 promoter were used as the template to transcribe biotin-labeled RNA by using T7 RNA polymerase (Thermo Fisher Scientific) in the presence of biotin-UTP (Biotium), 1 μg purified biotin-labeled transcripts were incubated with cell lysates for 30 min at room temperature and then mixed with Dynabeads M-280 Streptavidin (Invitrogen). After washing thoroughly, the beads were analyzed by LC-MS/MS or Western blot.

### Mass spectrometry

The proteins obtained from sucrose density centrifugation or RNA pulldown were stacked in SDS-PAGE gel and stained with Coomassie blue R-250. The gel contained proteins were digested with Trypsin. Trypsin-digested peptides were analyzed by a Waters Xevo G2 Q-TOF LC-MS/MS System at The State Key Laboratory of Natural and Biomimetic Drugs (SKLNBD).

### RNA immunoprecipitation (RIP) and cross-linking and immunoprecipitation (CLIP) assay

RIP and CLIP assays were performed as previously described.^65,66^ Briefly, HepAD38 cells and Huh-7 cells transfected with prcccDNA and pCMV-Cre were lysed with RIP lysis buffer. For CLIP assays, the cells were exposed to 400 mJ/cm^2^ 254 nm ultraviolet light before lysate to enhance the binding capacity between the protein and RNA, and the lysate was treated with RNase I (Invitrogen). The cell lysates were incubated with TIAR antibody (BD Biosciences) or mouse IgG (Proteintech) embedded ProteinA beads at 4 °C overnight. After being washed five times, the beads were resuspended using NT2 buffer with RNase-free DNase I (Roche) and proteinase K (TransGen Biotech). The coprecipitated RNAs were isolated by using phenol-chloroform extraction and ethanol precipitation, and detected by quantitative real-time PCR.

### HBV infection

HBV inoculum (genotype D, subtype ayw) was prepared from HepAD38 cell supernatants by PEG8000 (merck) precipitation as previously described.^3^ The HepG2-NTCP cells were additionally supplemented with 4 μg/mL doxycycline (DOX, Merck) for 4 days to induce expression of NTCP, and cultured in HCM for 24 h. Then, cells were incubated with HBV inoculum in the presence of 4 μg/mL DOX, 4% PEG8000 and 2% DMSO (sigma) for 24 h.

### Ribo-seq

Ribo-seq was performed by Gene Denovo Biotechnology Co. (Guangzhou, China) as described previously.^67^ Briefly, HepAD38 cells were pretreated with a final concentration of 100 μg/mL cycloheximide (CHX) (sigma) for 2 min. Pretreated HepAD38 cells were lysed with lysis buffer and treated with RNase I (NEB) and 6 μL of DNase I (NEB) for 45 min at room temperature with gentle mixing. RFs were purified and used to generate deep sequencing libraries, which were sequenced using Illumina HiSeq^TM^ X10. In this study, to avoid interference from ORFs in other HBV transcripts, especially HBs ORF, we chose 2307–2846 region which is specific for Pol and Cp translation. To further analyze the translation efficiency of Pol, we used RiboWave to inferring the P-site of each RNA fragment, which helped us to identify the translation of overlapping Cp and Pol ORFs.^31^

### Supplementary information

Supplementary Materials

Supplementary Table 1: List of 120 host proteins detected by MS that might be associated with nucleocapsids

Supplementary Table 2: List of 150 host proteins detected by MS that might interact with pgRNA in HepG2 cells

## Data Availability

Raw files for Ribosome profiling sequencing data are deposited at the Sequence Read Archive under accession number PRJNA913568. Raw files for proteomics data are deposited at iprox under accession number IPX0005657000. Any additional information in this paper is available from the corresponding author upon request.
