# Supplementary material for: RNA binding protein TIAR modulates HBV replication by tipping the balance of pgRNA translation
Source: Signal Transduct Target Ther. 2023 Sep 13;8:346. doi: 10.1038/s41392-023-01573-7 (PMC10497612; doi:10.1038/s41392-023-01573-7)
Supplement: Supplementary file 1 — Supplementary Materials [file 41392_2023_1573_MOESM1_ESM.docx]

Supplementary Materials for

RNA binding protein TIAR modulates HBV replication by tipping the balance of pgRNA translation

Ting Zhang^1#^, Huiling Zheng^1#^, Danjuan Lu^1#^, Guiwen Guan^1^, Deyao Li^1^, Jing Zhang^1^, Shuhong Liu^2^, Jingmin Zhao^2^, Ju-Tao Guo^3*^, Fengmin Lu^1,4*^, Xiangmei Chen^1*^

1 Department of Microbiology and Infectious Disease Center, School of Basic Medical Sciences, Peking University Health Science Center, Beijing 100191, China.

2 Department of Pathology and Hepatology, The Fifth Medical Center of Chinese PLA General Hospital, Beijing 100039, China.

3 Department of Experimental Therapeutics, Baruch S. Blumberg Institute, Doylestown, Pennsylvania 18902, United States of America.

4 Beijing Key Laboratory of Hepatitis C and Immunotherapy for Liver Diseases, Peking University Hepatology Institute, Peking University People's Hospital, Beijing 100044, China.

# Ting Zhang, Huiling Zheng, and Danjuan Lu contributed equally and should be regarded as co-first authors

*****Correspondence to: Xiangmei Chen([xm_chen6176@bjmu.edu.cn](mailto:xm_chen6176@bjmu.edu.cn)), Fengmin Lu([lu.fengmin@hsc.pku.edu.cn](mailto:lu.fengmin@hsc.pku.edu.cn)), Ju-Tao Guo(ju-tao.guo@bblumberg.org)

**This PDF file includes:**

Supplementary Methods

Figures. S1 to S8

Tables S3 to S5

**Other Supplementary Materials for this manuscript include the following:**

Tables S1 to S2

Materials and Methods

**Cell cultures and transfection**

Huh-7 cells (Chinese Academy of Sciences), HepG2 cells (ATCC), HepAD38^1^, and HepG2-NTCP (gifts from professor Ningshao Xia) were cultured in the Dulbecco's modified Eagle medium (Gibco) supplemented with 10% fetal bovine serum (FBS) (Gibco), 100 IU/mL penicillin and 100 μg/mL streptomycin (Gibco) at 37 °C in a 5% CO2 atmosphere. HepAD38 cells were additionally supplemented with 400 µg/mL G418 sulfate (Amerrosco). All plasmid transfections in cells were performed using Lipofectamine 2000 (Invitrogen). The transfection of siRNA targeting TIAR (siTIAR) or siRNA control (siNC) was performed using Lipofectamine RNAiMAX Transfection Reagent (Invitrogen) by following the manufacturer’s instructions.

**Plasmids**

The prcccDNA and pCMV-Cre plasmids were gifts from Professor Qiang Deng^2^. The overexpression plasmid of TIAR (pCDH-TIAR-Flag) was constructed by inserting TIAR cDNA into the pCDH-Flag vector, which can express wild-type TIAR proteins with 3×Flag-tag in the C terminal. The pcDNA3.1-pgRNA-P-flag plasmid and the plasmid deleting bulge and loop structures were constructed by Shanghai Generay Biotech corporation. The pCMV-core, pCMV-HBx, pCMV-preS1-flag, and pCMV-preS2-flag plasmids were constructed by inserting DNA fragments obtained from PCR by HBV plasmid into the pCMV vector. The pcDNA3.1-Polymerase-flag plasmid was constructed by inserting Polymerase DNA fragment obtained from PCR by HBV plasmid into the pCMV vector.

**Western blot**

Cells were lysed in ice-cold M-PER Mammalian Protein Extraction Reagent (Thermo Fisher Scientific) containing cocktail protease inhibitors (Roche) and phosSTOP (Roche). Then cell lysates were centrifuged at 12000 rpm for 10 min at 4 °C and collected supernatants. Protein concentrations were determined by the BCA Protein Assay Kit (Thermo Fisher Scientific). Protein lysates were separated on SDS-PAGE gels (Thermo Fisher Scientific) and transferred to PVDF membranes (Millipore). The membranes were blocked and probed with antibodies. To measure the effect of HBV replication on TIAR localization, nuclear and cytosolic extracts were separately collected by NE-PER Nuclear and Cytoplasmic Extraction Reagents (Thermo Fisher Scientific). The gray intensities of WB bands were analyzed by ImageJ.

**Quantitative reverse transcription PCR (RT-qPCR)**

Total RNA was extracted from the cells using Trizol (Invitrogen) and reversely transcripted to cDNA by Transcriptor First Strand cDNA Synthesis Kit (Roche). RT-qPCR analysis was performed in Roche 480 using LightCycler 480 SYBR Green I Master (Roche). β-actin gene was used as an internal control.

**Time‐resolved fluoroimmuno assay (TRFIA)**

TRFIA was performed as manufacturer's instructions of the diagnostic kit for the quantitative determination of HBsAg or HBeAg (PerkinElmer). Briefly, 100 μL supernatant of cells was added into 96-well plates of detective kit, and oscillated at 700 rpm for 40 min at room temperature. After being washed four times, the plates were added DTTA-Eu and oscillated at 700 rpm for 40 min at room temperature. Then the plates were washed six times and added enhancement solution. The plates were oscillated at 700 rpm for 50 min at room temperature and detected by Time-resolved fluorescence analyzer (PerkinElmer). The concentration of HBsAg and HBeAg was calculated by using a standard curve.

**Immunofluorescence staining and RNA fluorescence in situ hybridization (FISH)**

After being washed 3 times with PBS, cells were fixed with 4% paraformaldehyde at room temperature for 15 min. Then cells were permeabilized with ice-cold methyl alcohol for 20 min and blocked in 5% goat serum at room temperature for 1 h. Cells were incubated with the primary antibody at 4 °C overnight. After being washed 3 times with PBS, cells were then incubated with the secondary antibodies at room temperature for 1 h, then stained with DAPI. RNA-FISH was performed as described previously^3^. The previously reported probe targeting the 5′ end of pgRNA was used (Sangon Biotech)^4^. At last, cells were examined under a TCS-SP8 STED confocal laser scanning microscope (Leica) or an inverted fluorescent microscope (Leica).

**Northern blot**

Total RNA was extracted from the cells using Trizol (Invitrogen). The Northern blot was performed as manufacturer’s directions. HBV RNA was resolved by electrophoresis in agarose gel and transferred onto nylon membranes. The 28S/18S rRNA in gel served as a loading control were detected in UV-light. The membrane was probed with digoxigenin (DIG)-labeled plus‐strand specific full‐length HBV riboprobe, followed by incubation of anti-DIG (Roche).

**Immunohistochemistry**

The paraffin blocks of liver tissues with HBV infection were sectioned at 5 mm and dewaxed, rehydrated and stained for standard IHC. Anti-TIAR antibody was used for detection. Digital images were captured using the Aperio Scan Scope GL Slide Scanner (Aperio Technologies). The histologic features of these digital images were analyzed using the Visio pharm software.

**Reference:**

1 Ladner, S. K. *et al.* Inducible expression of human hepatitis B virus (HBV) in stably transfected hepatoblastoma cells: a novel system for screening potential inhibitors of HBV replication. *Antimicrob Agents Chemother*. **41**, 1715-1720, (1997).

2 Qi, Z. *et al.* Recombinant covalently closed circular hepatitis B virus DNA induces prolonged viral persistence in immunocompetent mice. *J Virol*. **88**, 8045-8056, (2014).

3 Haimovich, G. & Gerst, J. E. Single-molecule Fluorescence in situ Hybridization (smFISH) for RNA Detection in Adherent Animal Cells. *Bio Protoc*. **8**, e3070, (2018).

4 Yao, Y. *et al.* RNA-Binding Motif Protein 24 (RBM24) Is Involved in Pregenomic RNA Packaging by Mediating Interaction between Hepatitis B Virus Polymerase and the Epsilon Element. *J Virol*. **93**, (2019).


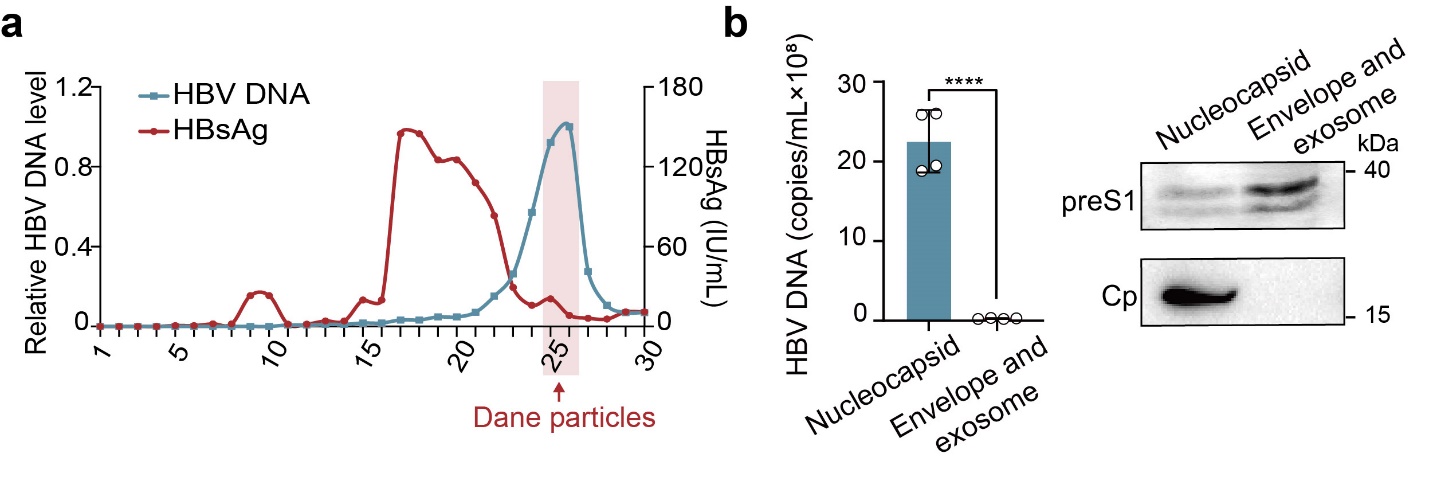


Figure. S1.

**Purification of nucleocapsids.** (a) The supernatant of HepAD38 cells cultured in the absence of Dox was subjected to PEG precipitation and sucrose density gradient centrifugation. The levels of HBV DNA and HBsAg in each fraction were detected by qPCR and time-resolved fluoroimmune assay (TRIA), respectively. (b)The fractions 25-26 were lysed and separated by centrifugation. The levels of HBV DNA (left), preS1 and core proteins (right) in the supernatants and pellets were detected by qPCR and Western blot, respectively. Data are represented as mean ± SD (n=4 per group), *****P* < 0.0001.


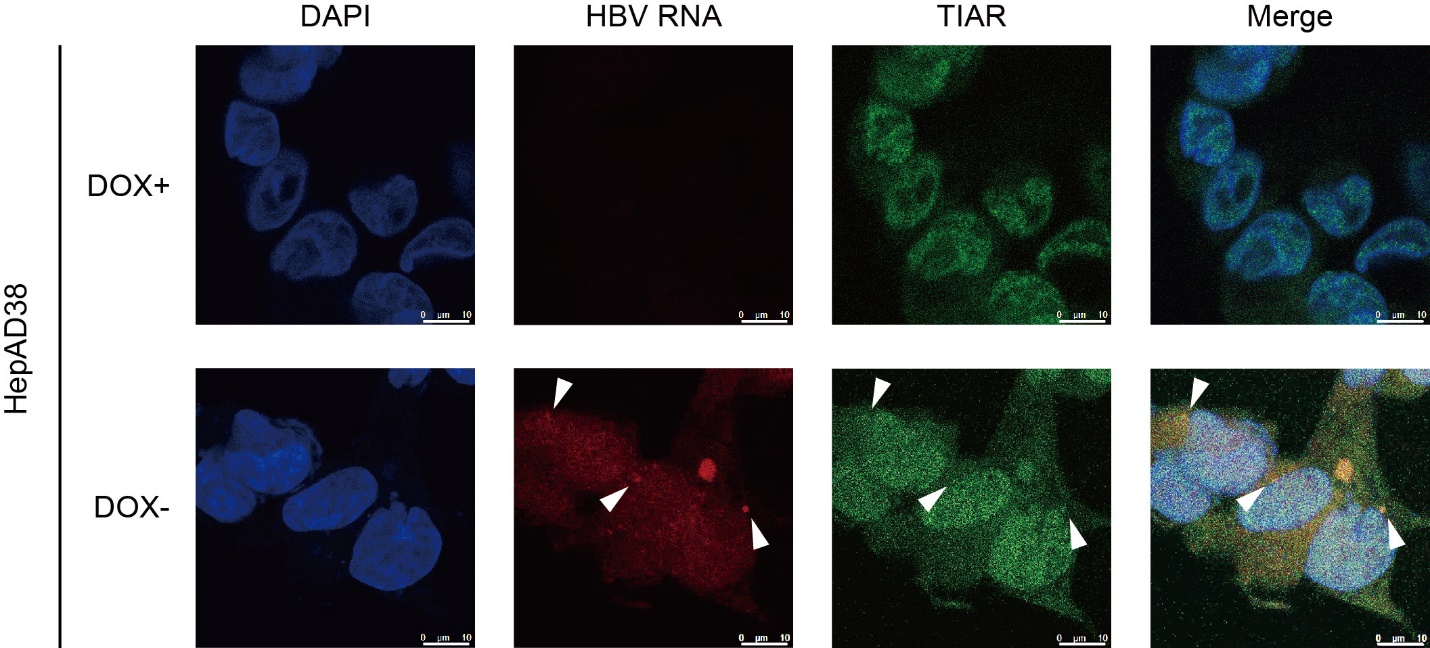


Figure. S2.

**TIAR colocalized with HBV RNA.** HepAD38 cells were fixed and subjected to RNA FISH and IF staining. A probe targeting the 5’ end of HBV RNA was used for HBV RNA staining. Nucleus were stained with DAPI. Scale bars: 10 µm.


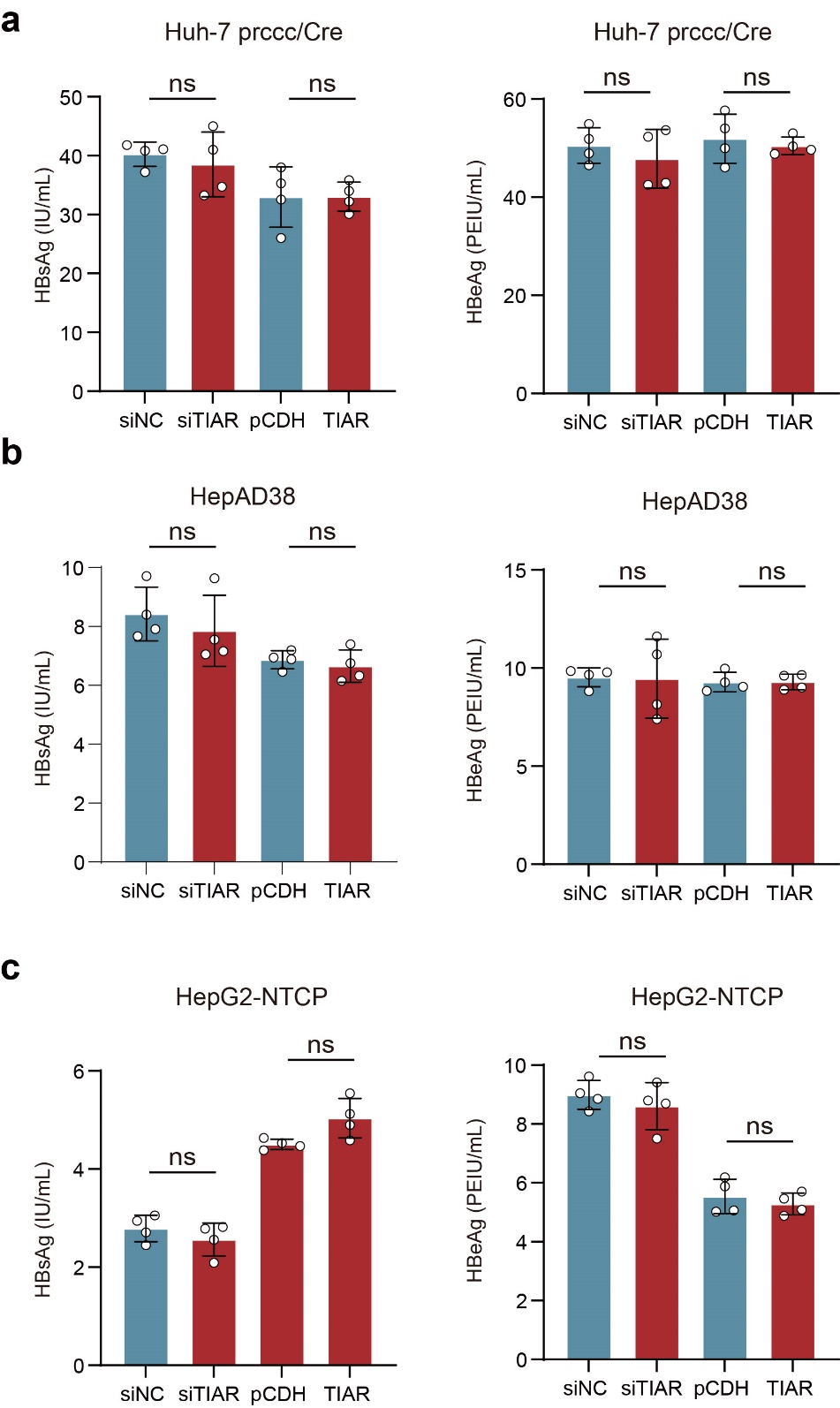


Figure. S3.

**Experimental modulation of TIAR expression does not alter the levels of secreted HBsAg and HBeAg in supernatants.** Huh-7 cells transfected with prcccDNA / PCMV-Cre plasmids (a), HepAD38 cells (b) and HBV infected HepG2-NTCP cells (c) were transfected with siTIAR or pCDH-TIAR-flag. The culture supernatant was harvested at 48 h post siRNA transfection. HBsAg and HBeAg in the supernatants were measured by a time-resolved fluoroimmuno assay (n=4 per group). Data are represented as mean ± SD, ns, no significance.


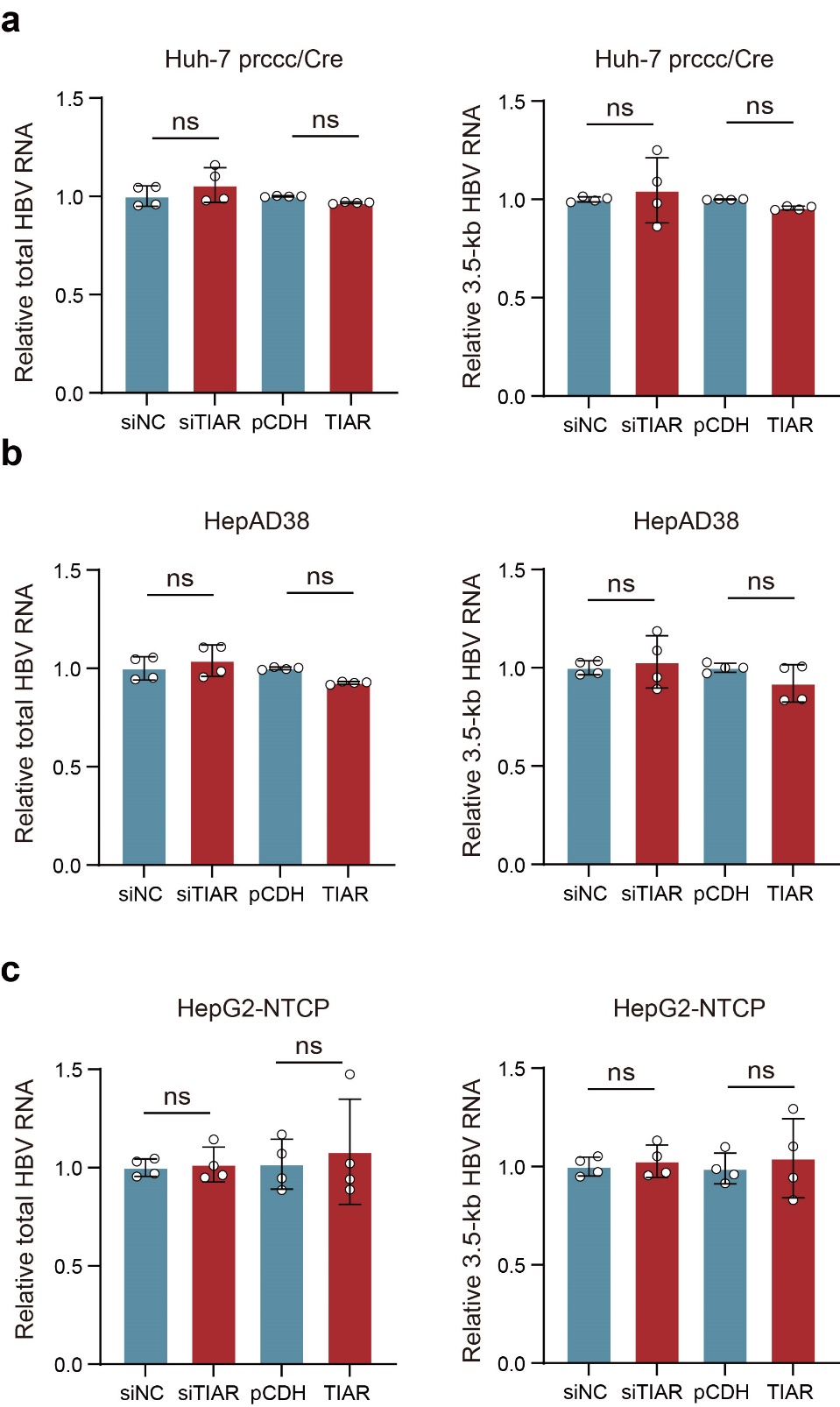


Figure. S4.

**Experimental modulation of TIAR expression does not alter the levels of intracellular HBV RNA.** Huh-7 cells transfected with prcccDNA / PCMV-Cre plasmids (a), HepAD38 cells (b) and HBV infected HepG2-NTCP cells (c) were transfected with siTIAR or pCDH-TIAR-flag. The cells were harvested at 48 h post siRNA transfection. The intracellular total and 3.5-kb HBV RNA were quantified by qRT-PCR assays (n=4 per group). Data are represented as mean ± SD, ns, no significance.


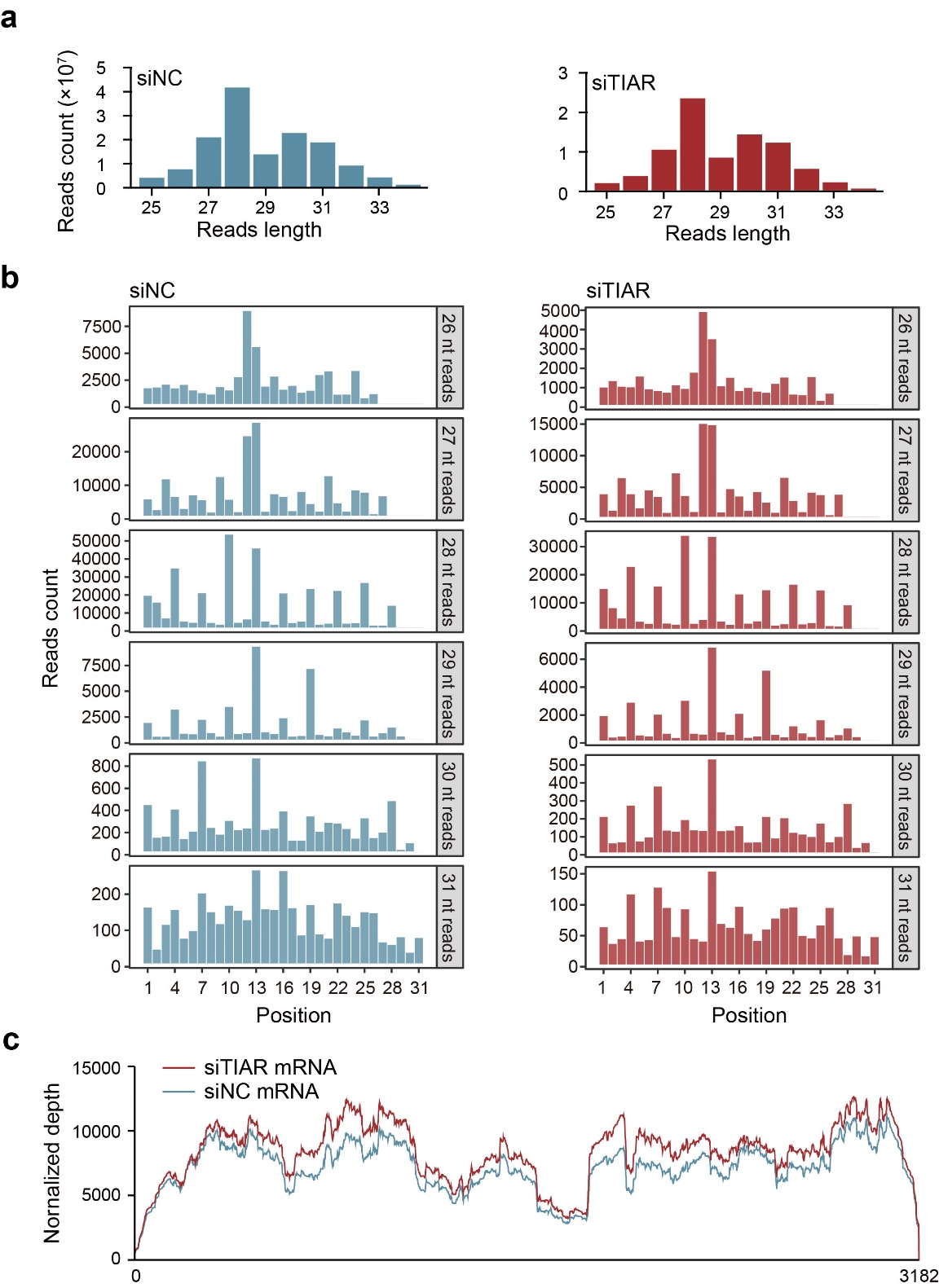


Figure. S5.

**Quality control of Ribo-seq.**  (a) The length distribution of RFs in HepAD38 cells transfected with siTIAR or siNC. (b) three‐nucleotide periodicity in HepAD38 cells transfected with siTIAR or siNC. (c) HBV mRNA levels in HepAD38 cells transfected with siTIAR or siNC detected by RNA-seq.


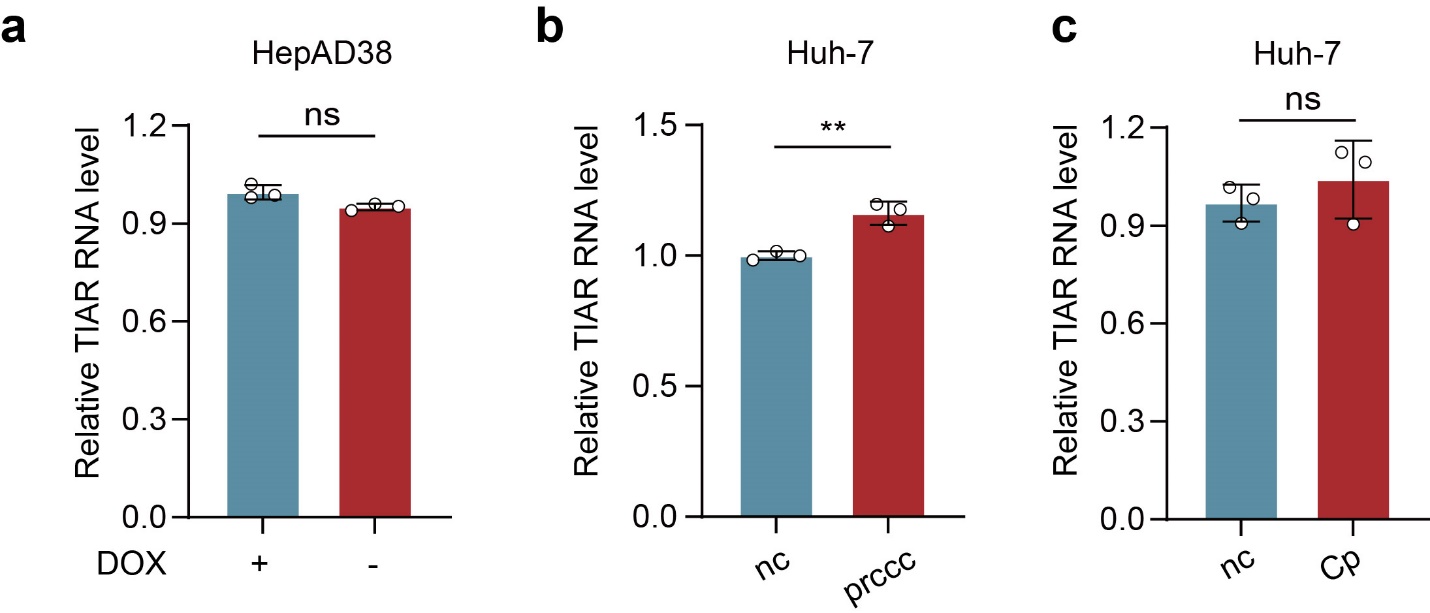


Figure. S6.

**HBV replication and core protein expression had no significant effect on TIAR RNA and the degradation of TIAR.** (a) In HepAD38 cells continuously cultured with Doxycycline and HepAD38 cells with the removal of Doxycycline for 11 days, TIAR RNA levels were detected by qRT-RCR assay. (b) Huh-7 cells were transfected with prcccDNA/ PCMV-Cre recombinant plasmids or empty vectors, intracellular TIAR RNA levels were determined by qRT-RCR assay. (c) Huh-7 cells were transfected with pCMV-Cp, TIAR RNA levels were measured by qRT-PCR assay (n=3 per group). Data are represented as mean ± SD, ***P*<0.01, ns, no significance.


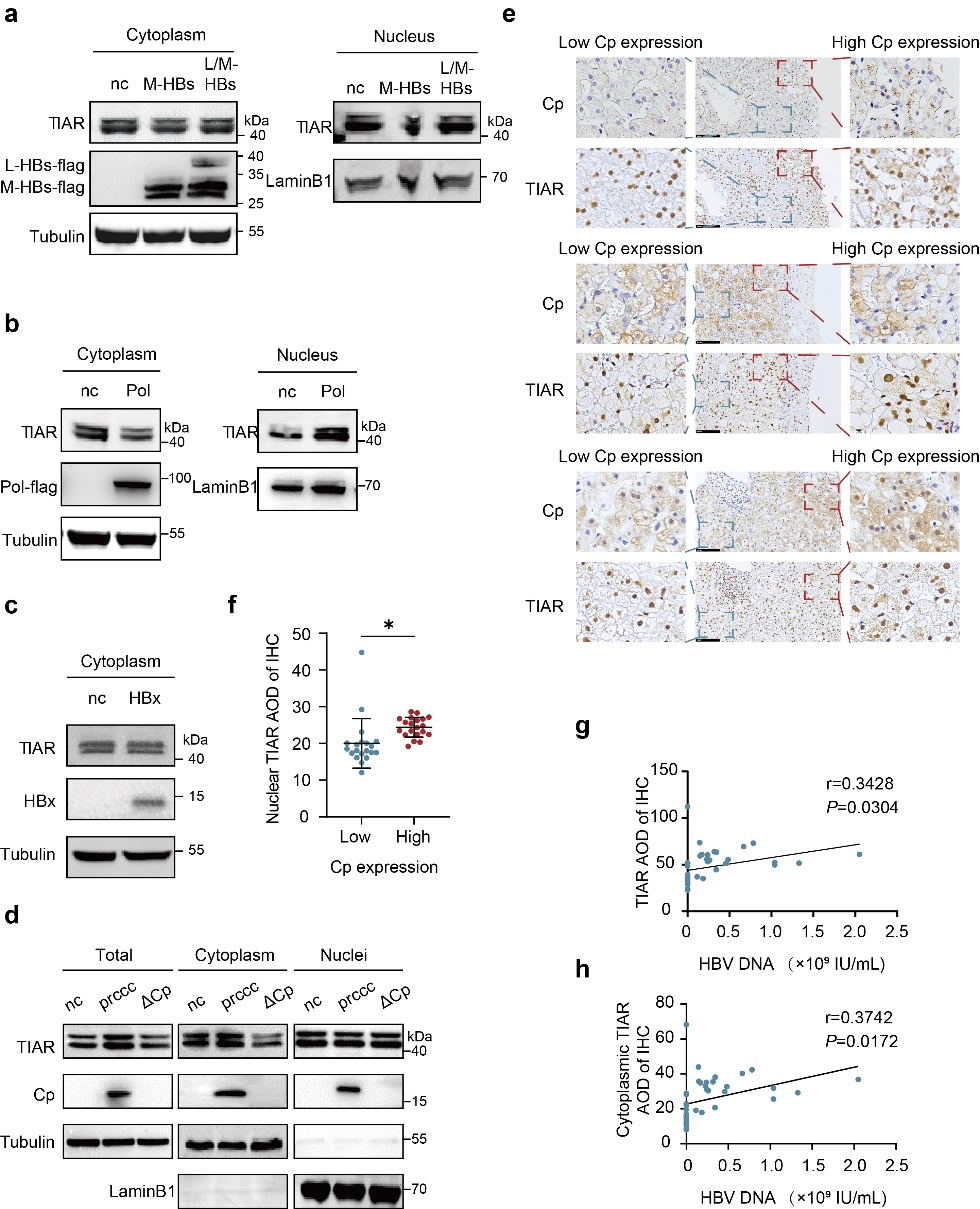


Figure. S7.

**The effect of individual HBV proteins on the subcellular distribution of TIAR.** (a) Huh-7 cells were transfected with pCMV-preS1-flag, pCMV-preS2-flag or vector, TIAR protein levels in the cytoplasm and nuclei were detected by Western blot. α-Tubulin and LaminB1 served as the marker of the cytoplasm and nuclei marker, respectively. (b) Huh-7 cells were transfected with pcDNA3.1-Pol-flag or vector. TIAR protein levels in the cytoplasmic and nuclear fractions were detected by Western blot. α-Tubulin and LaminB1 served as the maker of the cytoplasm and nuclei, respectively. (c) Huh-7 cells were transfected with pCMV-HBx or vector. Intracellular TIAR protein levels in the cytoplasm were determined by Western blot. α-Tubulin served as a loading control. (d) TIAR protein levels in the cytoplasm and nucleus were detected by Western blot in Huh-7 cells transfected with WT or mutant prcccDNA / pCMV plasmids. (e) TIAR subcellular localization in cells with different Cp expression in the same sample. The top, middle, and bottom images are liver biopsy samples from three patients. Scale bar: 100 μm. (f) Nuclear TIAR AOD value of 40 liver biopsy samples analyzed by ImageJ (n=20 per group). Data are represented as mean ± SD, **P*<0.05. (g) Correlation analysis between viral load and TIAR AOD. (h) Correlation analysis between viral load and cytoplasmic TIAR AOD.


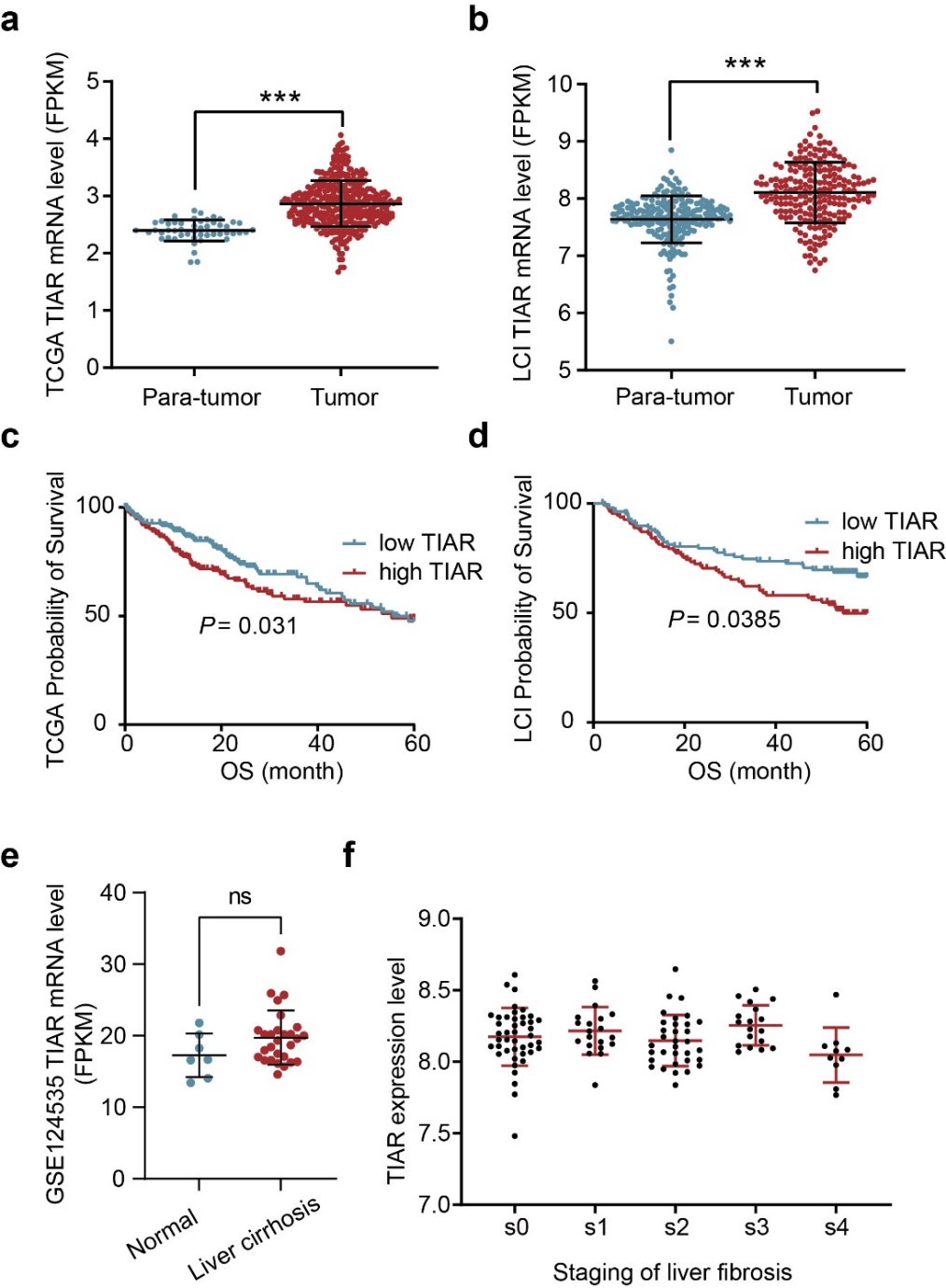


Figure. S8.

**The relationship between TIAR and HCC or liver cirrhosis.** (a) Relative TIAR mRNA level in the liver tissues of normal and HCC patients from TCGA database. (b) Relative TIAR mRNA level in the liver tissues of normal and HCC patients from LCI database. (c) Survival proportions of low or high TIAR levels from TCGA database. (d) Survival proportions of low or high TIAR levels from LCI database. (e) Relative TIAR mRNA level in the liver para-tumor tissues of HCC patients with or without liver cirrhosis from GSE124535 dataset. (f) Relative TIAR expression level in different staging of liver fibrosis from GSE84044 dataset. Data are represented as mean ± SD, ****P*<0.001, ns, no significance.

Table S1.

**List of 120 host proteins detected by MS that might be associated with nucleocapsids**

Table S2.

**List of 150 host proteins detected by MS that might interact with pgRNA in HepG2 cells**

Table S3.

**Clinical background of the studied patients**

|  | High Cp expression  (n=20) | Low Cp expression  (n=20) |
| --- | --- | --- |
| sex (F:M) | 1:1 | 1:1 |
| age (years) | 39.5 (19-53) ^a^ | 42 (21-61) ^a^ |
| BMI | 23.84 (16.85-29.66) ^a^ | 23.78 (16.72-30.77) ^a^ |
| ALT | 34 (10-411) ^a^ | 16 (11-90) ^a^ |
| HBV DNA (log_10_ copies/mL) | 8.51 (8.06-9.31) ^a^ | 2.52 (1.69-2.95) ^a^ |
| HBcAg IOD/area | 0.0128 (0.0065-0.0575) ^a^ | 1.9E-05 (0-0.0033) ^a^ |

^a^Median (range). F, female; M, male; ALT, alanine aminotransferase; IOD, Integrated Optical Density.

Table S4.

**Sequence of primers and target sequence of siRNA used in this study**

| **primer** | **Sequence (5’-3’)** |
| --- | --- |
| HBV DNA-F | CGGCGTTTTATCATMTTCCTCT |
| HBV DNA-R | GAGGACAAACGGGCAACATAC |
| HBV RNA-PreC-F | TCTGCGCACCAGCACCATG |
| HBV RNA-PreC-R | CAATGCTCAGGAGACTCTAAGGC |
| HBV RNA-3.5 kb-F | AGACCACCAAATGCCCCTATC |
| HBV RNA-3.5 kb-R | TCTGCGAGGCGAGGGAGTTC |
| HBV RNA-S-F | TGGCCAAAATTCGCAGTCCC |
| HBV RNA-S-R | GAAGAACCAACAAGAAGATGAGGC |
| HBV RNA-X-F | CATGGARACCACCGTGAACG |
| HBV RNA-X-R | CCAATTTATGCCTACAGCCTCCT |
| TIAR-qPCR-F | TGGTTGGGTGGTCGTCAAATC |
| TIAR-qPCR-R | CAGACGCAATTCCTCCACAGT |
| T7-HBV-F1-F | TAATACGACTCACTATAGGGATGGACATCGACCCTTATAAAG |
| T7-HBV-F1-R | ATTGAGACCTTCGTCTGCGAGGCGAGGGAG |
| T7-HBV-F5-F | TAATACGACTCACTATAGGGATGGCTGCTAGGCTGTGCT |
| T7-HBV-F5-R | TGAAAAAGTTGCATGGTGCTGGTGCGCAGA |
| T7-HBV-F3-F | TAATACGACTCACTATAGGGATGGGAGGTTGGTCTTCCAAA |
| T7-HBV-F3-R | AGGGTTCAAATGTATACCCAAAGAC |
| T7-HBV-F2-F | TAATACGACTCACTATAGGGCACCAAATGCCCCTATCTTATCAA |
| T7-HBV-F2-R | AACAGAAAGATTCGTCCCCATG |
| T7-HBV-F4-F | TAATACGACTCACTATAGGGGGGGGCCAAGTCTGTACAAC |
| T7-HBV-F4-R | CCATGGAAAGGAGGTGTATTTCC |
| T7-tRNA-F | TAATACGACTCACTATAGGGAAGGGCTTAGCTTAATTAAAGTGGCTGATTTGCGTTCAGTTGATGCAGAGTGGGGTTTTGCAGTCCTTA |
| T7-tRNA-R | TAAGGACTGCAAAACCCCACTCTGCATCAACTGAACGCAAATCAGCCACTTTAATTAAGCTAAGCCCTTCCCTATAGTGAGTCGTATTA |
| T7-HBV pgRNA-F | CGGATCAAACCGCACTGCTCTAATACGACTCACTATAGGGAGAAACTTTTTCACCTCTGCCTAATC |
| T7-HBV pgRNA-R | AGTTGAGGTATCCGTTGGTACCACAGTAGCTCCAAATTCTTT |
| CLIP-F1 | TCTGCGCACCAGCACCATG |
| CLIP-R1 | GACATGAACAAGAGATGATTAGGCA |
| CLIP-εF | ACCAGCACCATGCAACTTTTT |
| CLIP-εR | GATGTCCATGCCCCAAAGC |
| CLIP-F2 | ATAAAGAATTTGGAGCTACTGTGGA |
| CLIP-R2 | GAGGTGAACAATGCTCAGGAGAC |
| CLIP-F3 | CCTTCAGTACGAGATCTTCTAGATACC |
| CLIP-R3 | AATTAACACCCACCCAGGTAGC |
| CLIP-F4 | AGATCCAGCGTCTAGAGACCTAGTAG |
| CLIP-R4 | ACACTCCGAAAGACACCAAATACT |
| CLIP-F5 | AGACCACCAAATGCCCCTATC |
| CLIP-R5 | TCTGCGAGGCGAGGGAGTTC |
| CLIP-F6 | AATCTCGGGAATCTCAATGTTAGTAT |
| CLIP-R6 | CACATTTTTTGATAATGTCTTGGTGT |
| CLIP-F7 | AATTGATTATGCCTGCCAGGTT |
| CLIP-R7 | TCTTATATAATATACCCGCCTTCCAT |
| CLIP-F8 | CCATATTCTTGGGAACAAGATCTACA |
| CLIP-R8 | CGTCTGGCCAGGTGTCCTT |
| CLIP-F9 | AGGCTCAGGGCATACTACAAACTT |
| CLIP-R9 | AGTTTGGTGGAAGGTTGTGGAA |
| CLIP-F10 | GTGGCTCCAGTTCAGGAACAGTA |
| CLIP-R10 | AAGAAAAACCCCGCCTGTAA |
| CLIP-F11 | TGGCCAAAATTCGCAGTCCC |
| CLIP-R11 | GAAGAACCAACAAGAAGATGAGGC |
| CLIP-F12 | CTCAACAACCAGCACGGGAC |
| CLIP-R12 | CCGAAAGCCCAGGATGATG |
| CLIP-F13 | TTCTCCTGGCTCAGTTTACTAGTGC |
| CLIP-R13 | CGGTAAAAAGGGACTCAAGATGC |
| CLIP-F14 | AAACCCTAACAAAACAAAGAGATGG |
| CLIP-R14 | AGGCCTGTTAATAGGAAGTTTTCTAA |
| CLIP-F15 | TGTGGGTCTTTTGGGTTTTGC |
| CLIP-R15 | ACACTTGGCACAGACCTGGC |
| CLIP-F16 | CGATCCATACTGCGGAACTCC |
| CLIP-R16 | CCAGTTGGCAGCACAGCCTA |
| CLIP-F17 | CCTTTGTTTACGTCCCGTCG |
| CLIP-R17 | CGGTCCGGCAGATGAGAAG |
| CLIP-F18 | GCACGTCGCATGGAGACC |
| CLIP-R18 | GGTCGTTGACATTGCTGAGAGT |
| siTIAR-1 | GCAAGTACCGCCTTATGGA |
| siTIAR-1 | GGTCGTCAAATCCGAACCA |
| siTIAR-1 | CCAGAAAAGGGCTATTCAT |

Table S5.

**Antibodies message**

| Antibody | Source | Identifier |
| --- | --- | --- |
| TIAR (D32D3) XP Rabbit mAb | Cell Signaling Technology | Cat# 8509 |
| Purified Mouse Anti-TIAR | BD biosciences | Cat# 610352 |
| Mouse IgG antibody | Proteintech | Cat# B900620 |
| Anti-DDDDK-tag mAb | MBL International | Cat# M185-3 |
| Anti-α-Tubulin pAb | MBL International | Cat# PM054 |
| Anti-Hep B cAg Antibody (10E11) | Santa Cruz Biotechnology | Cat# sc-23947 |
| Fluorescein-Conjugated AffiniPure Goat Anti-Mouse IgG | Beijing Zhong Shan -Golden Bridge Biological | Cat# ZF-0312 |
| Mouse anti-core mAb | A gift from professor Ningshao Xia | |
| Mouse anti-core mAb | A gift from Beijing Hotgen Biotech Corporation | |
